# Age Dependence of Antimicrobial Resistance Among Fecal Bacteria in Animals: A Scoping Review

**DOI:** 10.3389/fvets.2020.622495

**Published:** 2021-01-26

**Authors:** Tara N. Gaire, Harvey Morgan Scott, Laura Sellers, T. G. Nagaraja, Victoriya V. Volkova

**Affiliations:** ^1^Department of Diagnostic Medicine/Pathobiology, College of Veterinary Medicine, Kansas State University, Manhattan, KS, United States; ^2^Department of Veterinary Pathobiology, College of Veterinary Medicine and Biomedical Sciences, Texas A&M University, College Station, TX, United States

**Keywords:** antimicrobial resistance, fecal bacteria, resistome, age dependence, cattle, swine, poultry, scoping review

## Abstract

**Introduction:** A phenomenon of decreasing antimicrobial resistance (AMR) among fecal bacteria as food animals age has been noted in multiple field studies. We conducted a scoping review to summarize the extent, range, and nature of research activity and the data for the following question: “does AMR among enteric/fecal bacteria predictably shift as animals get older?”.

**Methods:** This review followed a scoping review methodology framework. Pertinent literature published up until November 2018 for all animals (except humans) was retrieved using keyword searches in two online databases, namely, PubMed® and the Web of Science™ Core Collection, without filtering publication date, geographic location, or language. Data were extracted from the included studies, summarized, and plotted. Study quality was also assessed using the Grades of Recommendation, Assessment, Development, and Evaluation (GRADE) guidelines for all included papers.

**Results:** The publications with detailed relevant data (*n* = 62) in food animals, poultry, and dogs were identified. These included longitudinal studies (*n* = 32), cross-sectional studies of different age groups within one food animal production system or small-animal catchment area (*n* = 16), and experimental or diet trials (*n* = 14). A decline in host-level prevalence and/or within-host abundance of AMR among fecal bacteria in production beef, dairy cattle, and swine was reported in nearly two-thirds (65%) of the identified studies in different geographic locations from the 1970's to 2018. Mixed results, with AMR abundance among fecal bacteria either increasing or decreasing with age, have been reported in poultry (broiler chicken, layer, and grow-out turkey) and dogs.

**Conclusions:** Quantitative synthesis of the data suggests that the age-dependent AMR phenomenon in cattle and swine is observed irrespective of geographic location and specific production practices. It is unclear whether the phenomenon predates or is related to antimicrobial drug use. However, almost 50% of the identified studies predate recent changes in antimicrobial drug use policy and regulations in food animals in the United States and elsewhere.

## Introduction

Epidemiological studies in food animals have suggested that enteric antimicrobial resistance (AMR) changes as the host ages. For example, studies in beef and dairy cattle production systems in the Pacific Northwestern and Southwestern U.S. (1–3), Great Britain (4, 5), and Zambia (6) have shown that AMR gene copy abundance decreases in the fecal indicator bacterium *Escherichia coli* in cattle as animals age during early life. One of the studies conducted in the U.S. suggested that the decline in the abundance of multidrug-resistant (MDR) fecal *E. coli* in cattle during the first months of life may be independent of the transition from milk to solid diet (3). Other studies in pig production systems in the Midwestern U.S. showed that AMR gene copy abundance decreased in fecal *E. coli* in pigs during early life; in fact, the effect of age surpassed in relative magnitude to that of concurrent feeding with antimicrobial drugs (7). Others have reported that this decline in pigs continued beyond the first 2 weeks in the nursery, when the enteric microbiome changed due to the transition from milk to solid diet (8–10). Hence, the decline does not seem to be solely driven by the dietary transition of weaning. Understanding the dynamics of AMR is essential because, first, the body mass and fecal output of food animals increase with age (11). Thus, AMR among the fecal bacteria of larger older animals poses greater mass burden for AMR transmission *via* manure from production systems to the environment. Second, AMR among fecal bacteria at the age when the animal is harvested poses the greatest direct risk of AMR transmission to the consumer *via* carcass contamination at slaughter. Understanding the age-dependent AMR dynamics and their drivers could help lower both environmental and food safety risks.

Assessing the available research data pertaining to age-related AMR in food animals enhances our understanding of how the prevalence of animals carrying AMR bacteria or the abundance of AMR (or AMR genes) within those animals will change according to host age; in turn, this will help to formulate a risk-based AMR mitigation program to lower the environmental and food safety risks. However, it is unclear what kind of research information is available in the literature about age-dependent AMR in animals in different geographic regions of the world and across different periods. Thus, the objectives of this review were to examine research reports and summarize the available data for the following question: “does AMR among enteric/fecal bacteria predictably shift as animals get older?.” A scoping review was chosen as the approach for the study, given that the goal was to examine the nature, extent, and range of research activity on this broad question (12, 13) as well as to identify research gaps in the existing literature.

Here, we explore, summarize, and present data on age-related AMR dynamics from the available literature that was systematically searched for all animals without filtering date, geographic location, or the language of the publication. Using the Grades of Recommendation, Assessment, Development, and Evaluation (GRADE) guidelines (14), we summarized the quality of the evidence for all those identified studies containing relevant data. Moreover, following the scoping review approach, data from all the identified studies were charted and compiled. The outputs from the review results were summarized to directly address the study question.

## Materials and Methods

We followed the scoping literature review methodology framework outlined by Arksey and O'Malley (15). For rigor and reproducibility of the study, the review was implemented by adhering to the Preferred Reporting Items for Systematic Reviews and Meta-Analyses (PRISMA) guidelines (16, 17), more specifically, to the PRISMA extension for scoping reviews (PRISMA-ScR) (18) and for reporting purposes.

### Study Outcomes Relevant to the Study Question

The following outcomes were defined for “AMR among enteric/fecal bacteria” in a sampled animal population: the abundance of AMR bacteria, the relative fraction of AMR bacteria, the quantities of total or specific genes encoding AMR in feces of animals, and the proportion of animals carrying AMR bacteria or genes in their feces collected by individual fecal or rectal swabs or in a pooled sample from the pen/flock/barn. The study designs included any observational design employing a comparison group.

### Search Strategy

Keyword-based search strings were developed, refined, and implemented in two online databases: PubMed® maintained by the U.S. National Center for Biotechnology Information (NCBI) and Web of Science™ Core Collection maintained by the Thomson Reuters Corporation. The string for each database was refined until the search returned all publications known to the study team on the study question. The following final algorithms were used in PubMed®: (antibiotic resistance OR antibiotic resistant OR drug resistance OR multiple drug resistance OR resistance genes OR antimicrobial resistance OR antimicrobial resistant OR bacterial resistance) AND (fecal OR feces OR fecal OR feces OR stool OR intestinal OR intestine OR enteric OR bacteria OR bacterial OR fecal coliforms OR fecal coliforms OR fecal coliform OR fecal coliform OR coliform bacteria OR fecal flora OR fecal flora OR feces collection OR feces collection OR fecal examination OR fecal examination OR cecal OR caecal OR ceca OR caeca OR cecum OR caecum OR intestinal microorganism OR intestinal microorganisms OR *Enterobacteriaceae* OR *Escherichia coli* OR *E. coli* OR *Salmonella* OR *Campylobacter* OR *Enterococcus* OR *Klebsiella* OR *Citrobacter* OR microbial flora OR microbiome OR intestinal microorganisms) AND (age OR animals by age OR age groups OR age structure OR aging OR maturation OR cohort OR cohort studies OR longitudinal studies OR longitudinal distribution).

Similarly, the following final algorithms were used in the Web of Science™ Core Collection: (antibiotic resistance OR antibiotic resistant OR drug resistance OR multiple drug resistance OR resistance genes OR antimicrobial resistance OR antimicrobial resistant OR bacterial resistance) AND (fecal OR feces OR faecal OR faeces OR stool OR intestinal OR intestine OR enteric OR bacteria OR bacterial OR faecal coliforms OR fecal coliforms OR faecal coliform OR fecal coliform OR coliform bacteria OR faecal flora OR fecal flora OR feces collection OR faeces collection OR faecal examination OR fecal examination OR cecal OR caecal OR ceca OR caeca OR cecum OR caecum OR intestinal micro-organism OR intestinal microorganisms OR *Enterobacteriaceae* OR *Escherichia coli* OR *E. coli* OR *Salmonella* OR *Campylobacter* OR *Enterococcus* OR *Klebsiella* OR *Citrobacter* OR microbial flora OR microbiome OR intestinal microorganisms) AND (age OR animals by age OR age groups OR age structure OR aging OR maturation OR cohort OR cohort studies OR longitudinal studies OR longitudinal distribution) NOT (human OR man OR human disease OR human feces OR human stool OR children OR women OR infants).

Literature for all animals other than humans was searched (including farm, pet, or hobby animals and wildlife) without filtering the language, geographic location, or date of publication. The final searches were performed in November 2018. All citations identified by the searches in the two databases were imported, merged, and deduplicated in the web-based RefWorks^©^ v.2.0 platform (ProQuest, LLC, Ann Arbor, MI, USA). After deduplication, the citation list was imported into the web-based Rayyan platform for systematic reviews (19).

### Relevance Screening and Study Selection Criteria

The identified citations (*n* = 8,073) were subjected to a title-based screening. A citation was excluded if the title indicated that the study was conducted in humans, was performed on the resistance to disease of multicellular organisms, was on infectious agents other than enteric/fecal bacteria, or was on bacterial resistance to drugs other than antimicrobial drugs. The citations retained after the title screening (*n* = 383) were subjected to an abstract-based screening. A citation was excluded if the abstract met any of the title exclusion criteria. In addition, a citation was retained if the abstract identified that the study met these inclusion criteria: (a) performed in farm, shelter, or household animals; (b) longitudinal study of >3 weeks or a cohort study or a cross-sectional study of multiple age groups in one production system or local population (e.g., animals from multiple farms or owners); (c) enteric/fecal culturable bacteria or microbiome studied; and (d) AMR (phenotype or genes) measured in the bacteria or microbiome. The citations retained after the abstract screening (*n* = 199) were subjected to full-text screening. The full text was excluded if any of the abstract exclusion criteria were met or if it was an *in vitro* study or a cross-sectional study of animals at one age point or if the animal age was not specified. The full text was included if it met the abstract inclusion criteria, if the publication contained age-specific data on enteric/fecal AMR in animals of a given species (and in a given production system for food animals) at more than one age point, and either the fecal/enteric bacterial species was isolated and antimicrobial drugs to which the isolate susceptibility was tested were specified or the fecal/enteric bacterial antimicrobial genes tested were specified. The screening was performed on the Rayyan platform. These steps yielded 62 studies. The references for all included studies are provided in Supplementary Table 1.

Each title screening and abstract screening was performed independently by two reviewers. Citations on which the two disagreed were reviewed independently by a third reviewer whose judgment was the final decision. For refinement of the inclusion and exclusion criteria at the start of the title review, 50 titles were randomly selected from the citation list from PubMed® and independently reviewed following draft criteria by the first two reviewers. The three reviewers met to discuss the results and to clarify and refine the criteria. A similar criteria refinement procedure was performed at the start of the abstract review using 50 abstracts randomly selected from the citations (*n* = 383) retained after the title screening. The scoping review diagram was created following the PRISMA guidelines (16, 17) using the PRISMA-ScR (18). Given the difference in objectives from a systematic review, some PRISMA checklist items might not be relevant, while other important considerations may be missing; therefore, for transparent reporting purposes, we have provided a complete checklist of this PRISMA-ScR in Supplementary Table 2. In addition, an assessment of the quality of the final retained citations was made based on the GRADE assessment and risk-of-bias approach (14).

### Data Extraction, Characterization, and Analyses

The characteristics of each full text, such as year of publication, geographic location of the study, study design (longitudinal, experimental trial of antimicrobial drug use or diet, and cross-sectional), sample size for the fecal sampling, animal species, animal production system (e.g., dairy or beef), animal production category (e.g., broiler breeder or chicken), age or age group of the sampled animals, individual or pooled fecal sampling (e.g., from individual animals or pooled from multiple animals), sample type (e.g., rectal swab, cloacal swab, fecal grab per rectum, grab sample from voided feces), and AMR assessment method (e.g., testing phenotypic antimicrobial drug susceptibility of culturable bacterial species isolates, presence of AMR genes in culturable bacterial species, or AMR gene quantification *via* metagenomic sequencing of fecal samples), were recorded. For studies based on phenotypic AMR assessment, bacterial species isolated for testing the phenotypic susceptibility to antimicrobial drugs, number of isolates of each of the bacterial species, identity and class of each antimicrobial drug tested, percentage of the isolates of each bacterial species resistant to each individual antimicrobial drug, animal-level prevalence of AMR to each individual drug, percentage of the isolates of each bacterial species concluded to be MDR (≥3 drug classes), and animal-level prevalence of multidrug AMR were used in the study. For studies based on AMR gene quantification *via* metagenomics of fecal samples, the quantities of total and specific genes encoding bacterial AMR, identity and number of antimicrobial drug classes for which resistance is encoded by the detected genes, animal-level prevalence of the detected genes being carried in feces, and the total number of isolates were extracted from the full-text citations. Similarly, for the purpose of data extraction, studies published in non-English language were translated using an online translation tool. The above-described categories were not established *a priori* but were developed iteratively by reviewers and characteristics based on individual studies.

All the data captured above were recorded in Microsoft Excel 2010. The data charted in the scoping review were summarized by publication date (e.g., number of published studies each year), study location, study design, animal species, animal age, AMR analysis (phenotypic or genotypic), and fecal bacteria tested (e.g., *E. coli, Enterococcus* spp., *Salmonella* spp., *Campylobacter* spp.), etc. Similarly, extracted data from studies were aggregated based on the outcomes of interests (e.g., the proportion of animals carrying AMR bacteria or AMR genes, abundance or proportion of phenotypic AMR bacteria, or presence and number of AMR genes in feces of animals) and summarized by the age of animals. Age-dependent AMR dynamics (e.g., percentage of animal yielding AMR fecal *E. coli*) were further visualized by age animals (i.e., cattle, pigs, and poultry). The plotted individual observations represent AMR to particular drugs. Some studies recorded the age of animals as the stage of production cycle, for instance, piglet, nursery, grower, and finisher in the age data for pigs. To make the data consistent, data from such studies were categorized based on production stages (e.g., for pigs, piglet 1–3 weeks, weaner 3–4 weeks, nursery 4–10 weeks, grower 10–14 weeks, and finisher 14–26 weeks). We used R software with the ggplot2 package (V.3.3.4) (20) to summarize and visualize the data.

## Results

**Figure 1:**
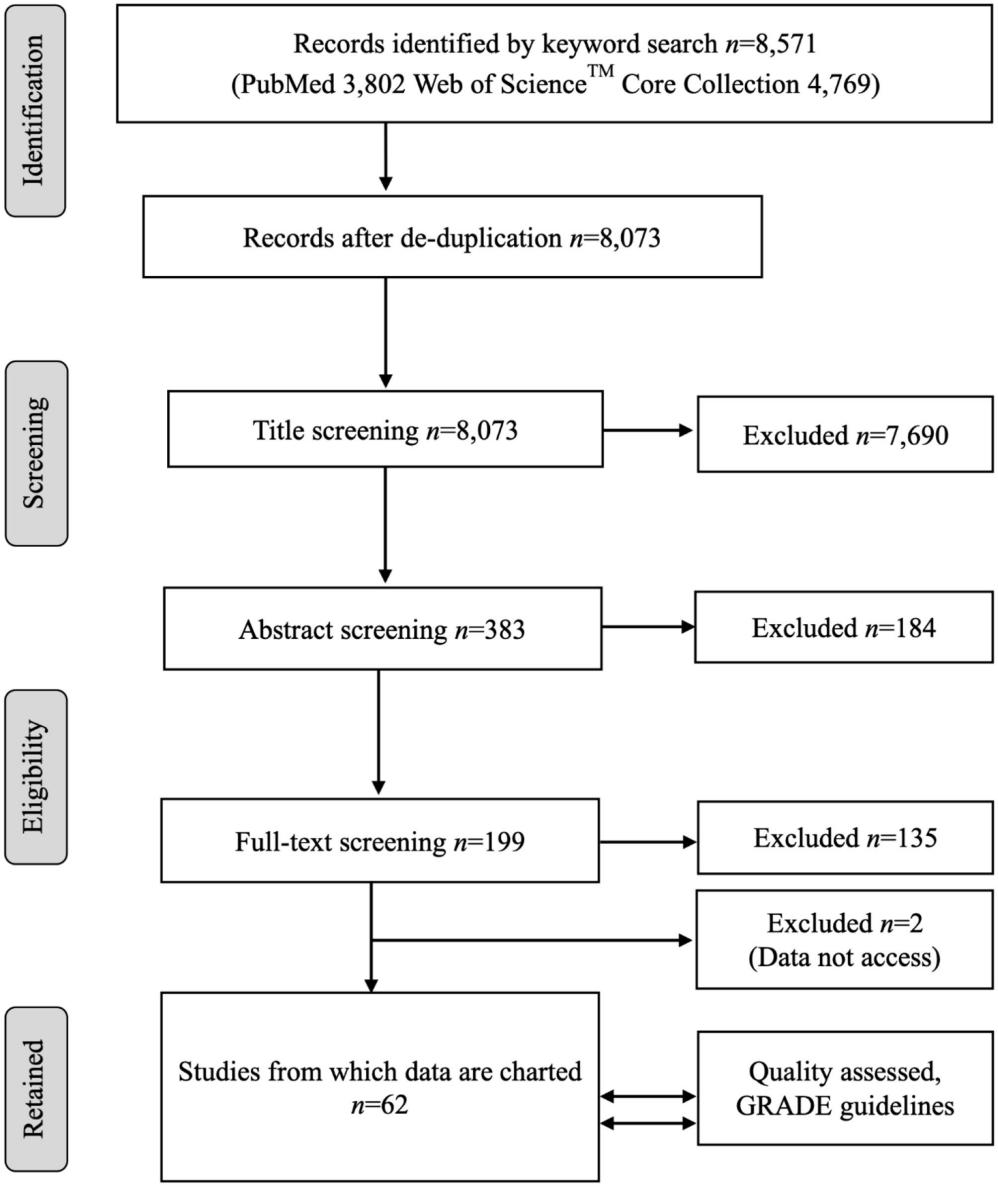
The PRISMA flow diagram of the scoping review of the literature on the age dependency of antimicrobial resistance of fecal bacteria in animals. *n*, number of studies.

A flow diagram of the process and the number of citations in the literature review is presented in Figure 1. The keyword searches in the NCBI PubMed database yielded 3,802 articles. The keyword search in the Web of Science™ Core Collection yielded 4,769 articles. The article sets were merged, and duplicate records were removed. This left 8,073 unique articles, the titles of which were screened by following the title exclusion criteria. After the removal of 7,690 titles, the abstracts of 383 records were reviewed and further screened based on the inclusion and exclusion criteria, and finally, 199 full articles were reviewed. After further exclusion, a total of 62 studies met the inclusion criteria, providing relevant information based on the review question, and were included for qualitative analysis. A list of all the studies included in this review is presented in Supplementary Table 1.

**Figure 2:**
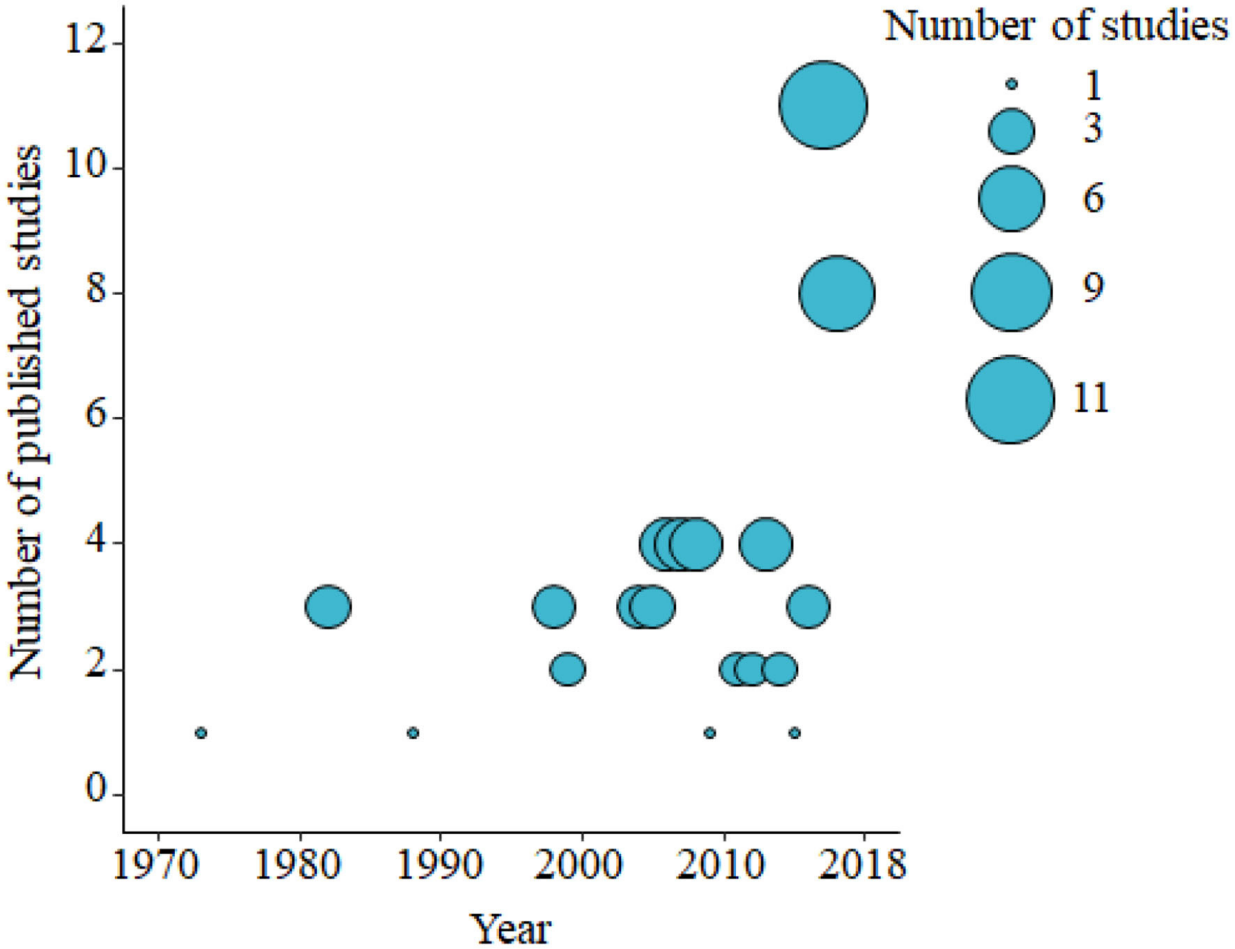
Bubble plot showing the number of published studies of age-dependent antimicrobial resistance in food animals by decade from the 1970's to 2010's (2000–November 2018). The bubble size is proportional to the number of studies in that decade. A total of 62 studies were identified and included in the analysis.

**Figure 3:**
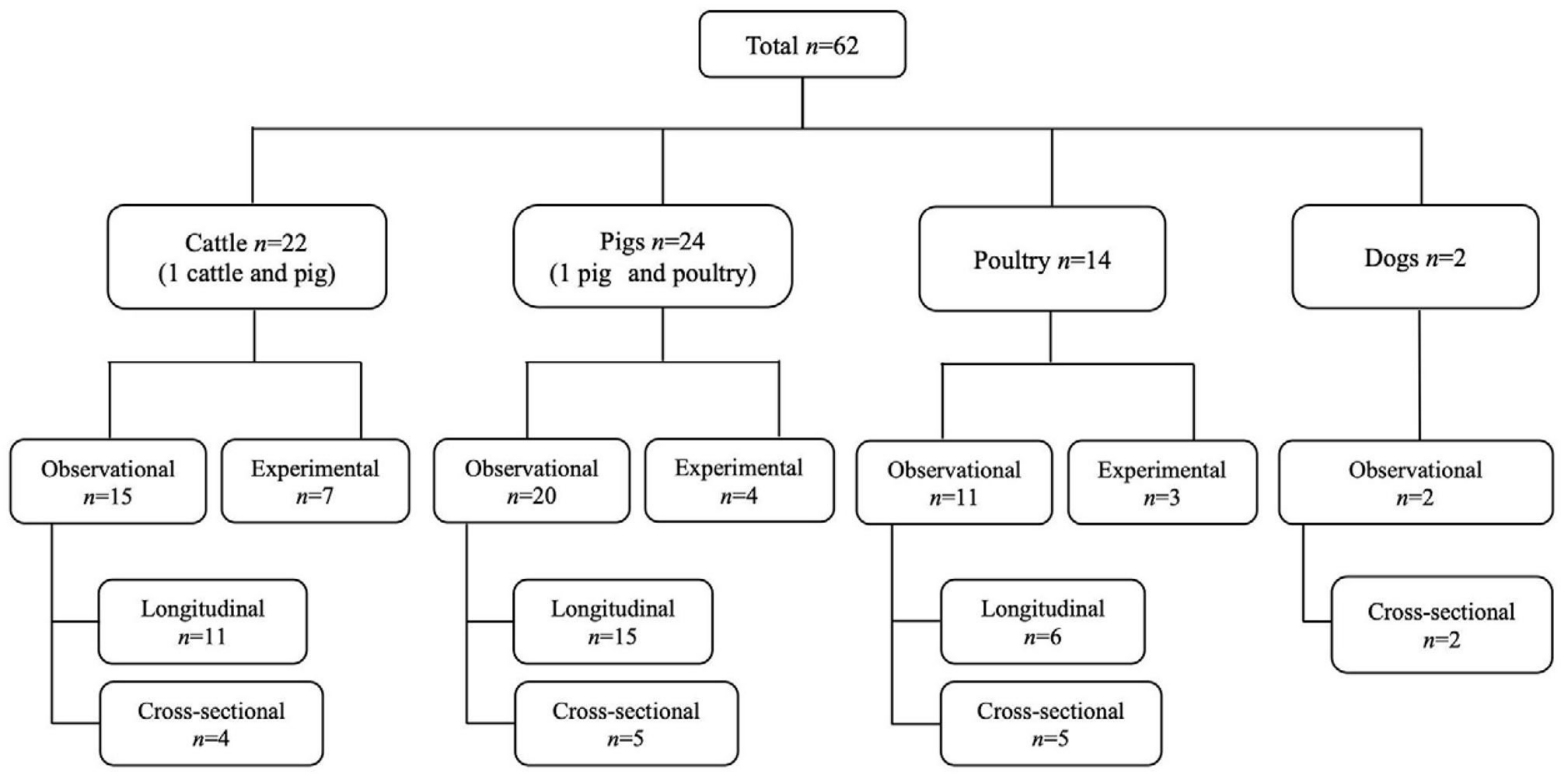
Distribution of the study design and animal species in the studies from which the data on the age dependency of antimicrobial resistance of fecal bacteria in animals were extracted in this review. *n*, number of studies.

**Figure 4:**
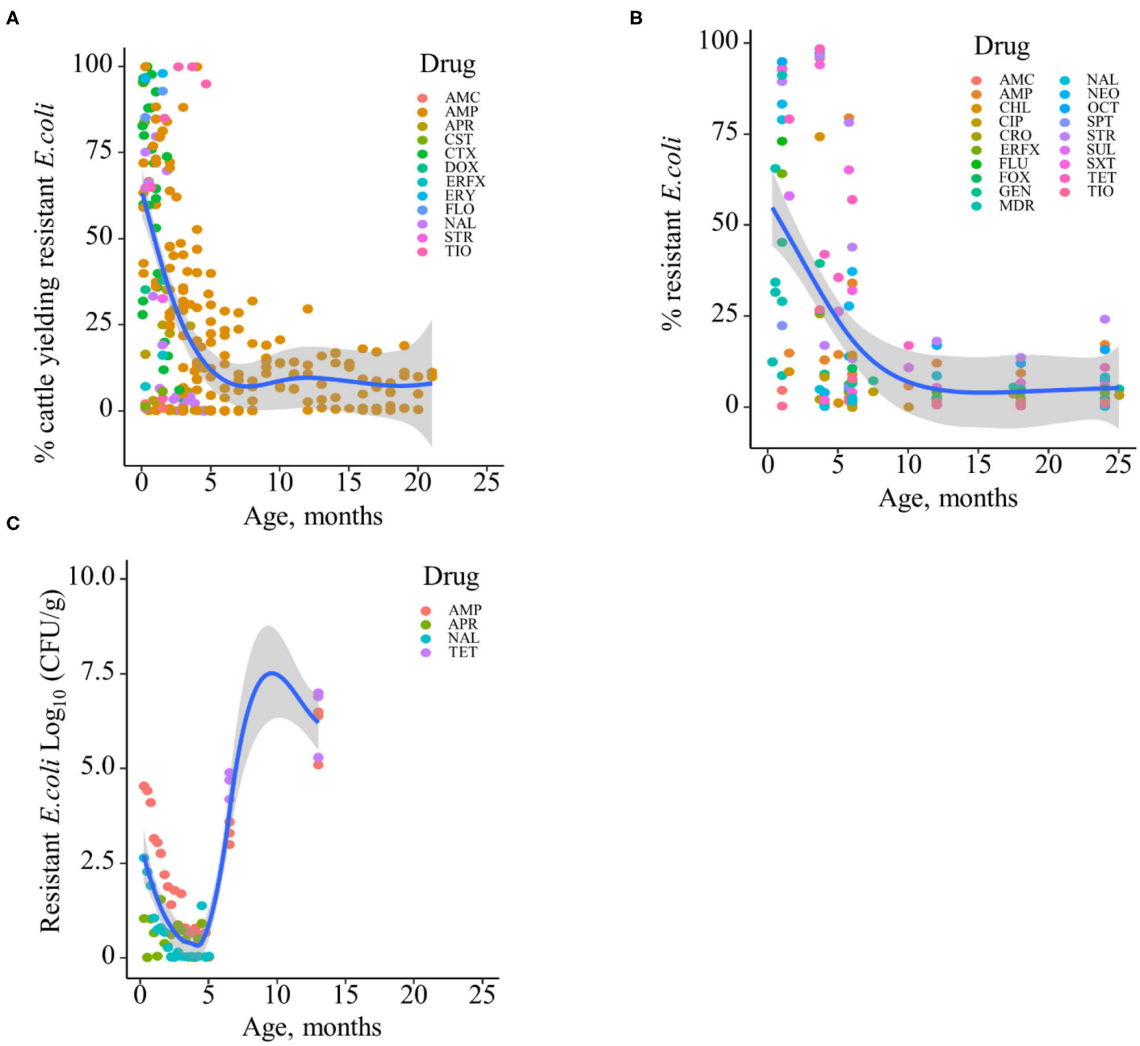
Age-dependent antimicrobial resistance dynamics in cattle. **(A)** Percentage of cattle yielding antimicrobial-resistant fecal *E. coli* by age (data from *n* = 10 studies). **(B)** Percentage of antimicrobial-resistant fecal *E. coli* by age (data from *n* = 7 studies). **(C)** Abundance of antimicrobial-resistant fecal *E. coli* (CFU/g) by age (data from *n* = 2 studies). Plotted individual observations represent antimicrobial resistance to individual drugs (AMC, amoxicillin–clavulanic acid; AMP, ampicillin; APR, apramycin; CHL, chloramphenicol; CIP, ciprofloxacin; CRO, ceftriaxone; CST, colistin; CTX, cefotaxime; DOX, doxycycline; ERFX, enrofloxacin; ERY, erythromycin; FLO, florfenicol; FLU, flumequine; FOX, cefoxitin; GEN, gentamicin; NAL, nalidixic acid; NEO, neomycin; OCT, oxytetracycline; SPT, spectinomycin; STR, streptomycin; SUL, sulfamethoxazole; SXT, trimethoprim–sulfonamides; TET, tetracycline; TIO, ceftiofur); MDR—multidrug resistance (to ≥3 drug classes). The blue trend lines with the confidence bands (the gray area around the blue line) are shown in **(A–C)**; these in each case were estimated using locally weighted scatterplot smoothing (LOESS).

**Figure 5:**
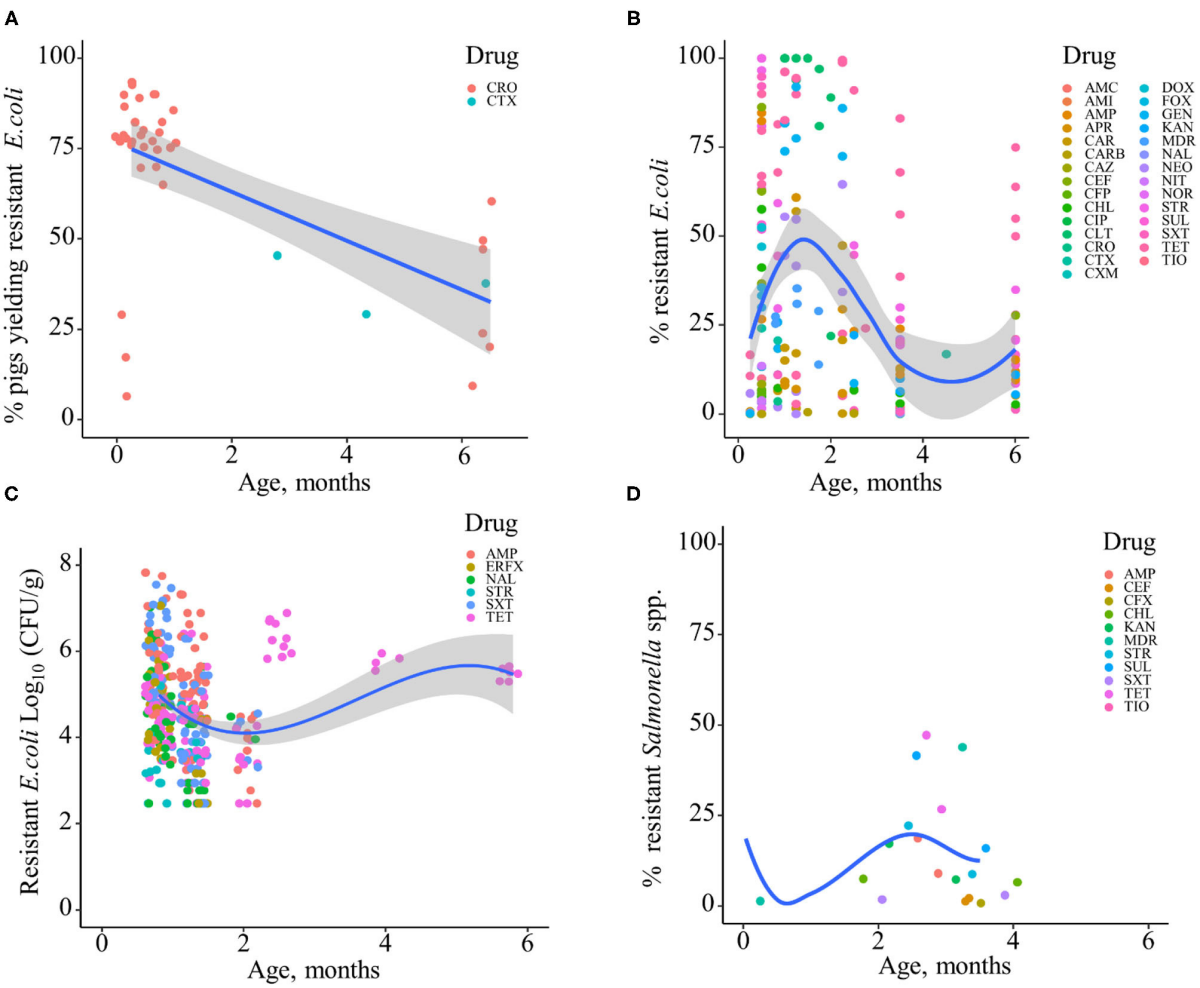
Age-dependent antimicrobial resistance dynamics in production pigs. **(A)** Percentage of pigs yielding antimicrobial-resistant fecal *E. coli* by age (data from *n* = 2 studies). **(B)** Percentage of antimicrobial-resistant fecal *E. coli* by age (data from *n* = 13 studies). **(C)** Abundance of antimicrobial-resistant fecal *E. coli* (CFU/g) (data from *n* = 2 studies). Plotted individual observations represent antimicrobial resistance to individual drugs (AMC, amoxicillin–clavulanic acid; AMI, amikacin; AMP, ampicillin; APR, apramycin; CAR, carbadox; CARB, carbapenem; CAZ, ceftazidime; CEF, cephalothin; CFP, cefoperazone; CHL, chloramphenicol; CIP, ciprofloxacin; CLT, chlortetracycline; CRO, ceftriaxone; CTX, cefotaxime; CXM, cefuroxime; DOX, doxycycline; ERFX, enrofloxacin; FOX, cefoxitin; GEN, gentamicin; KAN, kanamycin; NAL, nalidixic acid; NEO, neomycin; NIT, nitrofurantoin; NOR, norfloxacin; STR, streptomycin; SUL, sulfamethoxazole; SXT, trimethoprim–sulfonamides; TET, tetracycline; TIO, ceftiofur). MDR—multidrug resistance (to ≥3 drug classes). The blue trend lines with the confidence bands (the gray area around the blue line) are shown in **(A–C)**; these in each case were estimated using locally weighted scatterplot smoothing (LOESS). **(D)** Percentage of antimicrobial-resistant fecal *Salmonella* spp. (data from *n* = 4 studies).

**Figure 6:**
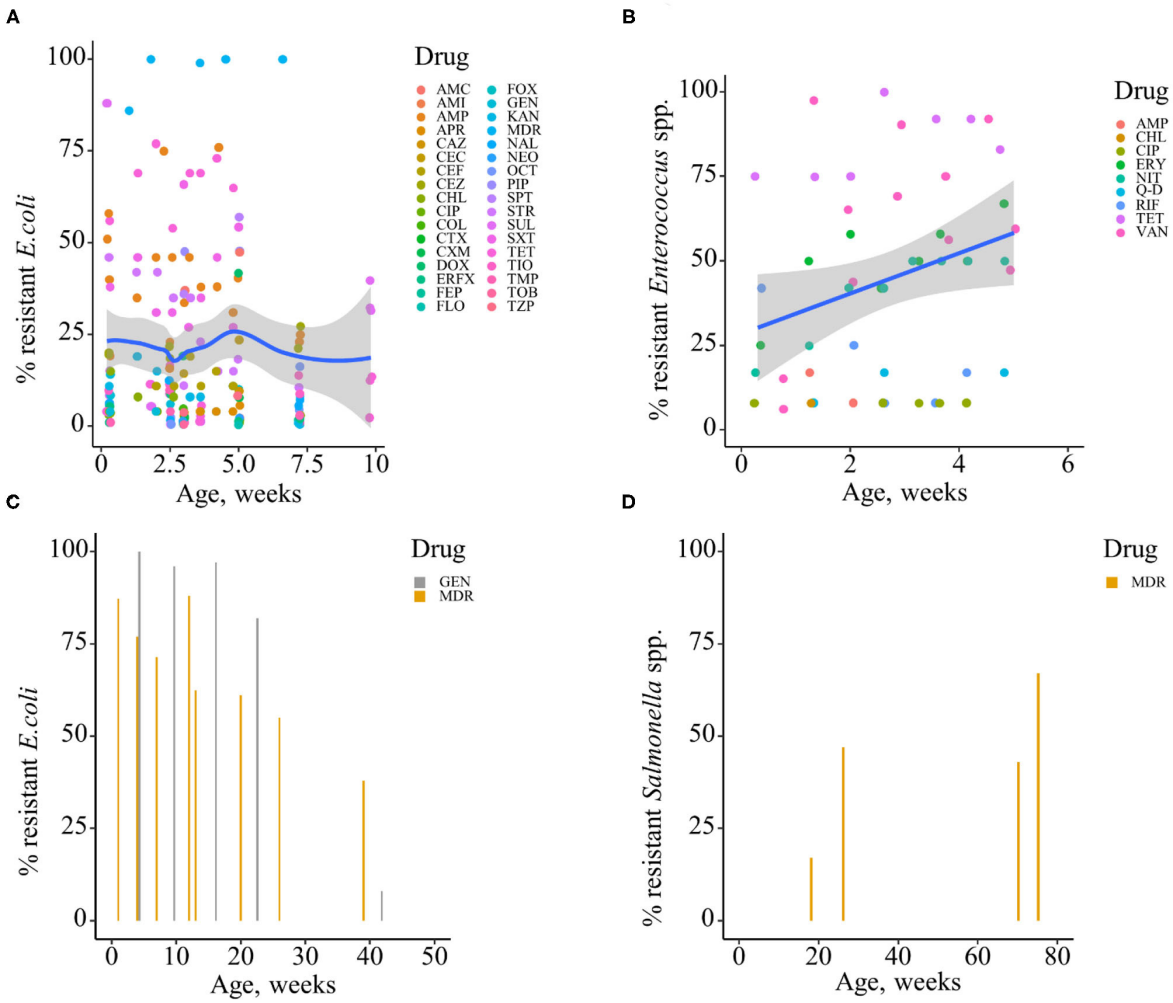
Age-dependent antimicrobial resistance dynamics in poultry (broiler, layer, and turkey). **(A)** Broiler chicken, percentage of antimicrobial-resistant fecal *E. coli* (data from *n* = 6 studies). **(B)** Broiler chicken, percentage of antimicrobial-resistant fecal *Enterococcus* spp. (data from *n* = 3 studies). **(C)** Turkey and layer, percentage of antimicrobial-resistant fecal *E. coli* (data from *n* = 2 studies). **(D)** Turkey and layer, percentage of antimicrobial-resistant fecal *Salmonella* spp. (data from *n* = 2 studies). Plotted individual observations represent antimicrobial resistance to individual drugs (AMC, amoxicillin–clavulanic acid; AMI, amikacin; AMP, ampicillin; APR, apramycin; CAZ, ceftazidime; CEC, cefaclor; CEF, cephalothin; CEZ, cefazolin; CHL, chloramphenicol; CIP, ciprofloxacin; COL, colistin; CTX, cefotaxime; CXM, cefuroxime; DOX, doxycycline; ERFX, enrofloxacin; FEP, cefepime; FLO, florfenicol; FOX, cefoxitin; GEN, gentamicin; KAN, kanamycin; NAL, nalidixic acid; NEO, neomycin; OCT, oxytetracycline; PIP, piperacillin; Q–D, quinupristin–dalfopristin; RIF, rifampicin; SPT, spectinomycin; STR, streptomycin; SUL, sulfamethoxazole; SXT, trimethoprim–sulfamethoxazole; TET, tetracycline; TIO, ceftiofur; TMP, trimethoprim; TOB, tobramycin; TZP, piperacillin–tazobactam); MDR—multidrug resistance (to ≥3 drug classes). The blue trend lines with the confidence bands (the gray area around the blue line) are shown in **(A,B)**; these in each case were estimated using locally weighted scatterplot smoothing (LOESS).

Based on the data gathered in this review, age dependency of AMR among fecal bacteria in animals has been reported since 1970. The absolute level of research activity peaked in 2010–2018 (*n* = 33, 53%) (Figure 2). Of the 62 studies, 30 (48%) were conducted in Europe, 22 (35%) in the U.S., 7 (11%) in Asia, and the remainder 3 studies (5%) in Canada. Among the 62 studies, only 2 studies were non-English languages. The animal populations studied in the retained citations (*n* = 62) were cattle (*n* = 22, including one with both cattle and pig), pigs (*n* = 24, including one with both pig and poultry), poultry (*n* = 14), and dogs (*n* = 2). The most common study type was observational (*n* = 48, 77%) (Figure 3). The most frequently investigated enteric/fecal bacteria in the retained studies were *E*. *coli* (*n* = 44, 71%), *Salmonella enterica* subsp. *enterica* (*n* = 7, 11%), and *Enterococcus* spp. (*n* = 6, 10%). All the studies were published as journal articles. The general characteristics of the retained articles are presented in Table 1, and details are presented in Supplementary Table 3. For the 22 cattle studies (including one with both cattle and pig), data outcomes were reported as: 41% of the studies reported AMR *E. coli*; 36% reported the abundance of AMR *E. coli*; 4% reported AMR *S. enterica*; 4% reported the relative abundance of AMR in bacterial taxa; 4% reported the presence of AMR genes in feces of animals; 4% reported the proportion of both the animals carrying both AMR *E. coli* and the abundance of AMR *E. coli*; and 4% reported the abundance of both AMR *E. coli* and AMR genes in feces of animals. Similarly, 24 pig studies (including one with both cattle and pig and another with both pig and poultry) generated 25 combined data points, where the outcome was reported as: 8% of the studies reported the proportion of animals carrying AMR *E. coli*; 50% reported the abundance or proportion of AMR *E. coli*; 16% reported the proportion of AMR *Salmonella* spp.; 4% reported the proportion of AMR *Campylobacter* spp.; 4% reported the proportion of AMR *Enterococcus* spp.; 12% reported the presence of AMR genes in feces of animals; and 8% reported both the abundance and the proportion of AMR *E. coli* and the presence of AMR genes in feces of animals. Similarly, from a total of 14 poultry studies and 1 study with both pigs and poultry, 15 data points were generated, where outcomes were reported as: 50% reported the abundance or proportion of AMR *E. coli*; 14% reported the proportion of AMR *S. enterica* subsp. *enterica*; 21% reported the proportion of AMR *Enterococcus* spp.; 14% reported the presence of specific genes encoding AMR in feces of animals; and 7% reported the abundance or proportion of both AMR *E. coli* and *Enterococcus* spp. We captured only two studies conducted in dogs, where outcomes were reported as: 50% reported the proportion of AMR *Enterococcus* spp.; and 50% reported the presence of specific genes encoding AMR in feces. Furthermore, we visualized the AMR dynamic data according to the age of animals. Based on the combined data, we observed that the overall age-related AMR dynamics (both phenotypic and genotypic) were especially high at a young age and thereafter declined with the age of animals among fecal *E. coli* in cattle (Figure 4) and pigs (Figure 5). However, mixed age-related AMR dynamics were observed in fecal *E. coli, Enterococcus* spp., and *S. enterica* subsp. *enterica* in poultry (including broiler chicken, layer, and meat-type turkey) (Figure 6) and fecal *Enterococcus* spp. in dogs. Furthermore, we also combined the age-related AMR data for other bacterial species to determine whether there were any discrepancies among bacterial species in terms of age-related AMR dynamics.

**Table 1:** Characteristics of included studies (*n* = 62) from which the data were charted in this scoping review of the literature on age dependency of antimicrobial resistance of fecal bacteria in animals.

**Characteristic**	**Number of studies**	**Percentage of *n* = 62 studies**
**Publication date**		
1970–1979	1	2%
1980–1989	4	6%
1990–1999	5	8%
2000–2009	19	31%
2010–2018 (November)	33	53%
**Study location**		
UK, continental Western Europe, Eastern Europe, and Russia	30	48%
USA	22	35%
Canada	3	5%
Asia	7	11%
**Study design**		
Longitudinal	32	52%
Cross-sectional	16	26%
Experimental (antimicrobial drug treatment or diet trial)	14	23%
**Animal species**		
Cattle^*^ (1 cattle and swine)	22	35%
Swine^**^ (1 swine and poultry)	24	39%
Poultry (chicken, turkey)	14	23%
Dog	2	3%
**Fecal sample**		
From individual animals	51	82%
Pooled from multiple animals	11	18%
**AMR identification**		
Phenotypic AMR in culturable fecal bacteria	34	55%
Genes encoding AMR in fecal metagenome or culturable bacteria	1	2%
Both phenotypic AMR and AMR genes	27	44%
**Fecal bacteria cultured**		
*Escherichia coli*	44	71%
*Salmonella* spp.	7	11%
*Campylobacter* spp.	1	2%
*Enterococcus* spp.	6	10%
*E. coli* and *Salmonella* spp.	1	2%
*E. coli* and *Enterococcus* spp.	1	2%
*Staphylococcus* spp.	1	2%
None, AMR genes in fecal microbiome analyzed	1	2%

* *Includes one study with both cattle and swine*.

** *Includes one study with both swine and poultry*.

In addition to phenotypic AMR, we also evaluated the combined data for AMR genes from cattle, pigs, poultry, and dogs from the included studies. The data showed that the overall abundance of genes encoding AMR to different drug classes among fecal *E. coli* or among the total genes is similar to the phenotypic AMR data, with especially high values at earlier ages or sampling points in cattle and pigs. The age-related AMR genes among fecal bacteria in pigs are presented in Supplementary Figure 1. Overall, two-thirds (65%, *n* = 46) of the included studies from cattle and pigs showed a high prevalence and abundance of antimicrobial-resistant enteric/fecal bacteria early in life that subsequently declined with age. In contrast, half of the included studies in poultry showed an increase in the prevalence and abundance of AMR in fecal or cloacal bacteria with age. In dogs, mixed AMR dynamics with age were reported in the identified studies.

### GRADE-Based Study Evaluation to Summarize the Research Activity (Quality Assessment)

To summarize the quality of research activity for the study question, an evaluation of the quality of evidence in the retained studies (*n* = 62) was performed by adapting the GRADE assessment of study quality and the risk-of-bias approach (14). In summary, nearly all studies (*n* = 60, 97%) clearly defined the study objectives and sampling procedures (e.g., description of participant/animal). However, the description of animal selection (inclusion/exclusion criteria for subject selection in the case of observational studies) was inadequate. In addition, very few studies (*n* = 6, 10%) estimated the sample size. A majority of the studies (*n* = 13, 93% of the experimental studies) clearly described the experimental group, but there were very few studies (*n* = 6, 43%) that performed randomization for treatment allocation. Nearly all the studies (*n* = 61, 98%) clearly defined the method to measure the outcomes. Finally, more than 50% of the studies described the potential biases and/or confounders and adjusted or explained the results in the outcome and analysis section. Specific definitions used for quality assessment are provided in Table 2. The details of the quality assessment criteria are presented in Supplementary Table 4. In addition to the GRADE assessment, we also found that most studies (*n* = 51, 82%) reported the source of funding in their studies.

**Table 2:** Descriptive summary of the quality assessment of the retained studies (*n* = 62) on age dependency of antimicrobial resistance of fecal bacteria in animals.

**Individual quality criterion**	**Number of studies**	**Percentage of studies**
**Study objectives clearly defined (*****n*** **=** **62)**		
Yes	60	97%
Unclear	2	3%
**Sampling method (animal selection) clearly described (*****n*** **=** **62)**		
Yes	59	95%
Unclear	3	5%
**Sample size estimation included (*****n*** **=** **62)**		
Yes	6	10%
No	56	90%
**Inclusion and exclusion criteria for the sampled animals stated (*****n*** **=** **48 observational studies)**		
Yes	15	31%
Partially	19	40%
No	14	29%
**Experimental groups (treatment and control) clearly defined for an experimental study (*****n*** **=** **14)**		
Yes	13	93%
Unclear	1	7%
**Sampling unit/animal randomly assigned to the treatment for an experimental study (*****n*** **=** **14)**		
Yes	6	43%
Unclear	8	57%
**The methods for AMR analysis in the study clearly specified (*****n*** **=** **62)**		
Yes	61	98%
Unclear	1	2%
**Potential biases or confounders listed and accounted for in the statistical analysis (*****n*** **=** **62)**		
Yes	36	58%
Unclear (not fully listed)	18	29%
Not reported	8	13%

*Yes, quality criteria met; No, quality criteria not met; Partially, not entirely met; Unclear, insufficient information to evaluate quality criteria; N/A, not applicable; n, number of studies*.

## Discussion

This review assessed the extent, range, and nature of available research activity and systematically and transparently charted the main characteristics of AMR among enteric/fecal bacteria according to host age in food animals, poultry, and pet dogs. We identified research reports relating AMR prevalence or quantity among fecal bacteria with the age of the animal since the 1970's in different geographic regions of the world, with a surge of research (over 50% of the papers) since 2010. Additionally, most of the studies were from Europe and North America. We believe that this distribution likely reflects the geographic location in which the phenomenon is frequently investigated.

The available evidence, including study findings, shows declining host-level prevalence and within-host abundance of AMR among fecal bacteria from early life in cattle and pigs to later production stages in the different production systems. Both observational and experimental field studies showed that the prevalence of AMR in fecal bacteria declined with the age of animals, and interestingly, this phenomenon has been observed over the decades across dispersed geographic locations and different production practices: from small-scale cattle farms in Tajikistan in 1971 (21) to housing of thousands of cattle in the Southwestern U.S. in the 2010's (2, 3) to cattle farms in Zambia (6) and pig farms in Canada (22) and the U.S. (23). This lends credibility to the notion that the phenomenon may not be driven solely by antimicrobial drug practices on farms. Similarly, healthy calves were reported to be rapidly colonized by antimicrobial-resistant *E. coli* shortly after birth and to shed MDR bacteria that are resistant to 9 and 10 antibiotics as early as 1 day of age (24). Furthermore, intestinal microbiomes are unique in younger calves compared with adult animals, favoring the survival of MDR bacteria (1). Additionally, age-related dynamics are not limited to animals and have also been reported in humans (25, 26). For instance, age-specific AMR among *E. coli* was reported in a human in the UK, where the abundances of *E. coli* resistant to amoxicillin, co-amoxicillin/clavulanic acid, ciprofloxacin, cephalexin, and extended spectrum beta-lactamase (ESBL)-producing *E. coli* were high at a young age and decreased with age, followed later by an increase (27).

On the other hand, increased AMR in *E. coli* was reported in beef cattle (28) and in *Enterococcus* spp. in pigs (29) for production systems in European countries that have dramatically altered antimicrobial drug use practices since the 1990's (30, 31). For example, in Belgium and other European countries, the use of avoparcin was banned in 1997. In Belgium, most surprisingly, the pigs in which the growth promoter was banned (sows) demonstrated the highest prevalence of vancomycin-resistant *Enterococci* (VRE) compared with piglets and finishers from later birth cohorts. Another finding from the same study was that the prevalence of VRE was higher in broilers than in layers of the same age group; importantly, avoparcin had been used in the past in broilers but never in layer chicks (29). In Denmark, higher tetracycline and sulfonamide resistance was observed in *Salmonella typhimurium* isolates from pigs as well as from a human following bans on growth promoters went into effect (30). Recently, analysis of the abundance and diversity of the fecal resistome in pigs and broilers in nine European countries found that resistome abundance and composition were very different in pigs and poultry, that is, with higher abundance in pigs but a more diverse resistome in poultry. However, functionally determined AMR genes were not associated with drug use, suggesting that some genes might be functional only in a specific host. Furthermore, the findings revealed that countries with similarity in antimicrobial use also exhibited the same general levels of AMR (32).

Antimicrobials have been used in livestock production for disease treatment, control, and prevention and to increase feed efficiency and growth performance since the early 1950's (33). However, prolonged exposure to antimicrobial concentrations might increase the risk of AMR development, which could subsequently be transferred to humans (34). Due to these concerns, several countries have already implemented regulations to restrict antimicrobials use. For instance, in 2006, the European Union (EU) banned the use of antibiotics for growth promotion purposes. In the U.S., since 2017, all label indications for antimicrobials as growth promoters have been removed (35). These antimicrobial use changes may lead to several adjustments in animal production practices and subsequent AMR phenomena at the animal and farm levels. It is unclear whether the age-dependent AMR phenomenon is related to the recent antimicrobial drug use policy and regulations in food animals in the U.S. and elsewhere. However, based on our review data, age dependency of AMR among fecal bacteria in animals has been reported since 1970, and nearly 47% of the studies were published before the 2010's. Similarly, our review findings showed that the dynamics of AMR associated with age in poultry were different from those in cattle and pigs. Both increased AMR (36–38) and decreased AMR (29, 39) among enteric bacteria were reported in the poultry production cycles. These results might be due to the blanket use of antimicrobials in feed or else due to the direct and close contact between birds carrying antimicrobial-resistant strains. We did not capture a large number of citations for age dependency of AMR in dogs; however, we observed mixed dynamics of AMR in the included studies.

Based on the results, the following question arises: “is this age-related phenomenon the result of previous or current antimicrobial exposure?.” For instance, a longitudinal study on calves demonstrated that there was no significant association between calves fed waste milk containing antibiotic residue or calves fed fresh milk in terms of the proportion of animals that shed CTX-M-positive *E. coli* during the 1- to 12-week age period (40). Similarly, other studies have shown that resistome richness decreased significantly during the feeding period (arrival and exit, ~32 weeks) of feedlots when they traced AMR in the feedlots; but at the same time, other resistome elements were detected against antimicrobials that are not approved for use in cattle production, suggesting that the relationship between antimicrobial use and AMR is not straightforward, and that the use of antimicrobials alone cannot directly explain the presence of AMR (41). It has also been concluded that the elevated AMR in early life in cattle is not of maternal origin but likely acquired during the first weeks of life (due to factors other than antimicrobials added to fresh milk, if any) (2, 3, 42). More extensive research on the sources of AMR in the young has been performed in humans. Human newborns carry diverse AMR genes in their enteric microbiome before receiving antimicrobial treatments, and such individuals also have been born to mothers not treated with antimicrobial drugs during the last trimester of pregnancy (43).

The purpose of this review was to extract essential information from the diverse body of work conducted to address the relevant research questions; scoping reviews typically do not assess the quality of the studies included in the review (15). Quality assessment to control the biases in research analyses other than systematic reviews is rarely applied and poorly reported in veterinary science (44). For instance, some have reported quality assessments in scoping reviews (13), whereas others did not report any quality assessment to identify the risk of biases (45–49). A rigorous scoping review of scoping reviews was conducted by Pham et al. (50) to examine the approach and consistency. In these reviews, quality assessment of the included studies was infrequent, and only 22.4% of the 335 scoping reviews performed quality assessment checks. However, evaluation of quality or risk of bias in the included studies is recommended (51). For this review, first, we adopted the PRISMA-ScR (18) for the purposes of transparency and reproducibility; second, we implemented quality assessment of all retained citations by adapting the GRADE assessment and the risk-of-bias approach (14) and summarized the data based on the quality criteria (Table 2); however, we did not remove any articles from the assessment. In doing so, our review was able to provide a complete overview of all available research reports related to a topic, as per the objectives of the scoping review, and at the same time, we became aware of the quality of the evidence from the research analysis.

There are some limitations to our scoping review. Although nearly all (97%) the studies clearly mention the research objectives and outcome and are from journal articles, the data were from different types of study designs, and different interpretative criteria for antimicrobial susceptibility testing may have affected the findings. Any citations that were not listed in one of the search databases were not captured by this study. Therefore, this is something that needs to be taken into consideration when interpreting the data of this review. Our scoping review has shown that the age-related dynamics in fecal/enteric AMR in food animals, including poultry and pet animal dogs, were consistent with other field study results. Although we do not fully understand the mechanism underlying the high AMR at early ages, it seems that the epidemiology of the age group of the animal population has been found to be an important factor in the quantification of AMR. The production of food animals may involve public health risks related to microbial exposure, and there are many opportunities for the bacteria carrying resistance determinants to enter the food chain, regardless whether the animal received antimicrobials or not. Age-dependent AMR study in food animals will improve our understanding of how the prevalence of AMR is within the commensal or host during their production phase. We believe that this age-dependent AMR data finding helps to stratify further individual risk based on the study area, allowing the potential targeting of surveillance (i.e., threshold-based action-driven monitoring) or any other intervention in specific animal age groups in the population (e.g., beef cattle or poultry). However, this review specifically aimed to summarize the research reports related to the age dependency of AMR, so many policy-related questions remain unanswered. The higher AMR observed at early ages is of concern, but there was no clear difference in animal age-specific trends between different antimicrobial agents, which could represent differences in antimicrobial use and, in turn, selection pressure. Well-researched areas of interest were identified in this review (for example, why higher AMR in fecal bacteria is observed at early ages in animals), and this finding could be extended to other food animals and to aquaculture, which will ultimately help with AMR risk categorization and planning for interventions to reduce environmental and foodborne risks to public health.

## Conclusions

The decline in the prevalence and abundance of AMR enteric/fecal bacteria with age in production pigs, beef, and dairy cattle has been reported since the 1970's in various geographic locations and in two-thirds of the included studies in our scoping review. In broiler chickens and meat turkeys, mixed AMR dynamics associated with age have been reported. We captured very few studies in dogs, where mixed AMR dynamics with age also were reported. We found that the age of animals could be one of the factors affecting both phenotypic and genotypic AMR; however, other management factors may influence the overall findings. Hence, identifying such risk factors associated with resistance in the different production phases of food animals and poultry is crucial, and such findings could guide judicious antimicrobial use. The scientific evidence from the existing studies in these areas is limited. Therefore, further longitudinal research into related AMR phenomena should be undertaken to better guide the interpretation of our findings.

## Data Availability Statement

The datasets generated during and analyzed for this study are available from the corresponding author upon request.

## Author Contributions

VV and HS conceived the study. TG, HS, and VV designed the study and its specific components. TG and LS performed the literature screening. TG and VV drafted the manuscript and developed the figures. HS, LS, and TN helped write the manuscript. All authors contributed to the article and approved the submitted version.

## Conflict of Interest

The authors declare that the research was conducted in the absence of any commercial or financial relationships that could be construed as a potential conflict of interest.

## Abbreviations

AMR: antimicrobial resistance
MDR: multidrug resistant
PRISMA: Preferred Reporting Items for Systematic Reviews and Meta-Analyses.
