# Supplementary material for: Age Dependence of Antimicrobial Resistance Among Fecal Bacteria in Animals: A Scoping Review
Source: Front Vet Sci. 2021 Jan 26;7:622495. doi: 10.3389/fvets.2020.622495 (PMC7870994; doi:10.3389/fvets.2020.622495)
Supplement: Supplementary file 1 [file Data_Sheet_1.pdf]

Supplementary material

Supplementary Table 1. List of references that were retained (*n*=62) in the scoping review

Species (no. of studies)	References	Comments
Cattle (*n*=22)	(1-9, 10*, 11-22)	*one study including both cattle and pig
Pig (*n*=24)	(23-40, 41*, 42-46)	*one study including both pig and poultry
Poultry (*n*=14)	(47-60)	
Dog (*n*=2)	(61, 62)	

Supplementary Table 2. Preferred Reporting Items for Systematic reviews and Meta-Analyses extension for Scoping Reviews (PRISMA-ScR) Checklist

SECTION	ITEM	PRISMA-ScR CHECKLIST ITEM	REPORTED ON PAGE #
**TITLE**			
**Title**	1	Identify the report as a scoping review.	1
**ABSTRACT**			
**Structured summary**	2	Provide a structured summary that includes (as applicable): background, objectives, eligibility criteria, sources of evidence, charting methods, results, and conclusions that relate to the review questions and objectives.	1
**INTRODUCTION**			
**Rationale**	3	Describe the rationale for the review in the context of what is already known. Explain why the review questions/objectives lend themselves to a scoping review approach.	2
**Objectives**	4	Provide an explicit statement of the questions and objectives being addressed with reference to their key elements (e.g., population or participants, concepts, and context) or other relevant key elements used to conceptualize the review questions and/or objectives.	2
**METHODS**			
**Protocol and registration**	5	Indicate whether a review protocol exists; state if and where it can be accessed (e.g., a Web address); and if available, provide registration information, including the registration number.	NA
**Eligibility criteria**	6	Specify characteristics of the sources of evidence used as eligibility criteria (e.g., years considered, language, and publication status), and provide a rationale.	3-4
**Information sources***	7	Describe all information sources in the search (e.g., databases with dates of coverage and contact with authors to identify additional sources), as well as the date the most recent search was executed.	3-4
**Search**	8	Present the full electronic search strategy for at least 1 database, including any limits used, such that it could be repeated.	2-3
**Selection of sources of evidence†**	9	State the process for selecting sources of evidence (i.e., screening and eligibility) included in the scoping review.	Figure 1

**Data charting process‡**	10	Describe the methods of charting data from the included sources of evidence (e.g., calibrated forms or forms that have been tested by the team before their use, and whether data charting was done independently or in duplicate) and any processes for obtaining and confirming data from investigators.	3-4
**Data items**	11	List and define all variables for which data were sought and any assumptions and simplifications made.	3-4
**Critical appraisal of individual sources of evidence§**	12	If done, provide a rationale for conducting a critical appraisal of included sources of evidence; describe the methods used and how this information was used in any data synthesis (if appropriate).	Table 2, Supplementary Table 4
**Synthesis of results**	13	Describe the methods of handling and summarizing the data that were charted.	4-6
**RESULTS**			
**Selection of sources of evidence**	14	Give numbers of sources of evidence screened, assessed for eligibility, and included in the review, with reasons for exclusions at each stage, ideally using a flow diagram.	Figure 1
**Characteristics of sources of evidence**	15	For each source of evidence, present characteristics for which data were charted and provide the citations.	Table 1, and Supplementary Table 3
**Critical appraisal within sources of evidence**	16	If done, present data on critical appraisal of included sources of evidence (see item 12).	Table 2 and Supplementary Table 4
**Results of individual sources of evidence**	17	For each included source of evidence, present the relevant data that were charted that relate to the review questions and objectives.	Table 1, Figure 3
**Synthesis of results**	18	Summarize and/or present the charting results as they relate to the review questions and objectives.	Table 1, Supplementary Table 3, Figure 2-6 and Supplementary Figure 1
**DISCUSSION**			
**Summary of evidence**	19	Summarize the main results (including an overview of concepts, themes, and types of evidence available), link to the review questions and objectives, and consider the relevance to key groups.	6-11
**Limitations**	20	Discuss the limitations of the scoping review process.	11

**Conclusions**	21	Provide a general interpretation of the results with respect to the review questions and objectives, as well as potential implications and/or next steps.	12
**FUNDING**			
**Funding**	22	Describe sources of funding for the included sources of evidence, as well as sources of funding for the scoping review. Describe the role of the funders of the scoping review.	12

Source: Tricco AC, Lillie E, Zarin W, O'Brien KK, Colquhoun H, Levac D, et al. PRISMA Extension for Scoping Reviews (PRISMA-ScR): Checklist and Explanation. Ann Intern Med. 169:467–473. doi: 10.7326/M18

Supplementary Table 3. Study characteristics of the individual studies included in the review (*n*=62)

Species (no. of studies)	Author name (last), year	Country	Studied animal age	Study design	Study type	Bacteria tested	Sample	AMR analysis
Cattle (*n*=22)	Mir et al., 2018(1)	USA	Birth-12 months	Longitudinal	Observational	*E. coli*	Fecal, individual	Phenotypic, genotypic
	Pereira et al., 2018(2)	USA	Birth-6 weeks	Trial	Experimental	Microbiota	Fecal, individual	Microbiome taxa
	Maynou et al., 2017(3)	Spain	6 weeks and 1 year	Trial	Experimental	*E. coli*	Fecal, individual	Phenotypic
	Adler et al., 2017(4)	Israel	≤ 4 months, 5-10 months, 11-24 months, ≥ 25 months	Cross-sectional	Observational	*E. coli*	Fecal, individual	Phenotypic
	Ohta et al., 2017(5)	USA	Sampling days (0, 4, 8, 14, 20, 26)	Trial	Experimental	*Salmonella* spp.	Fecal, individual	Phenotypic
	Hutchinson et al., 2017(6)	USA	110 days-6 months	Longitudinal	Observational	*E. coli*	Fecal, individual	Phenotypic, genotypic
	Catry et al., 2016(7)	Belgium	Calves (4 and 23 weeks), beef cattle (6-24 months), dairy cattle	Longitudinal	Observational	*E. coli*	Fecal, individual	Phenotypic
	Pereira et al., 2015(8)	USA	3-5 months and 14-19 months	Cross-sectional	Observational	*E. coli*, *Salmonella* spp.	Fecal	Phenotypic
	Brunton et al., 2014(9)	UK	Birth-13 weeks	Trial	Experimental	*E. coli*	Fecal, individual	Phenotypic, genotypic
	Mazurek et al., 2013(10)*	Poland	Cattle (beef and dairy); Pig (6 to 8 weeks)	Cross-Sectional	Observational	*E. coli*	Fecal, individual	Phenotypic, genotypic
	Watson et al., 2012(11)	UK	Day1-day 161	Longitudinal	Observational	*E. coli*	Fresh floor fecal samples	Phenotypic, genotypic
	Alexander et al., 2011(12)	Canada	Beef cattle sampling days (1-175 days)	Trial	Experimental	AMR genes	Fecal, individual	Genotypic

	Sharma et al., 2008(13)	Canada	6.5 months-11 months	Trial	Experimental	*E. coli*	Fecal, individual	Phenotypic, genotypic
	Berge et al., 2006(14)	USA	4 weeks	Trial	Experimental	*E. coli*	Rectal fecal samples, individual	Phenotypic
	Liebana et al., 2006(15)	UK	~ 1 months	Longitudinal	Observational	*E. coli*	Fecal, individual	Phenotypic, genotypic
	Hoyle et al., 2006(16)	UK	Calves (1-8 weeks); Cattle (≤30 months and ≥ 30 months)	Longitudinal	Observational	*E. coli*	Fecal, individual, and environmental	Phenotypic, genotypic
	Donaldson et al., 2006(17)	USA	2.5-19 weeks	Longitudinal	Observational	*E. coli*	Fecal, individual	Phenotypic, genotypic
	Berge et al., 2005(18)	USA	Birth-6 weeks	Longitudinal	Observational	*E. coli*	Fecal, individual	Phenotypic
	Hoyle et al., 2005(19)	UK	1-21 weeks	Longitudinal	Observational	*E. coli*	Fecal, individual	Phenotypic, genotypic
	Hoyle et al., 2004(20)	UK	Birth-21 weeks	Longitudinal	Observational	*E. coli*	Fecal, individual	Phenotypic
	Hoyle et al., 2004(21)	UK	1-8 months	Longitudinal	Observational	*E. coli*	Fecal, individual	Phenotypic
	Khachatryan et al., 2004(22)	USA	Pre-weaned calves	Cross-sectional	Observational	*E. coli*	Fecal, individual	Phenotypic, genotypic
Pig (*n*=24)	Ciesinsk et al., 2018(23)	Germany	24 days (one day before weaning), 38 days (two weeks after weaning), 52 days (four weeks after weaning)	Trial	Experimental	*E. coli*	Fecal, individual	Phenotypic
	Randall et al., 2018(24)	UK	8 weeks, 17 weeks, 24 weeks, 20 months	Longitudinal	Observational	*E. coli and mcr-1 gene*	Fecal, individual	Phenotypic, genotypic
	Mollenkopf et al., 2018(25)	USA	Piglets to Finisher	Longitudinal	Observational	*Enterobacteriaceae/bla*$_{\text{IMP-6}}$	Fecal, Individual	Genotypic

Cameron-Veas et al., 2018(26)	Spain	Day 7, 9, 14 and 187	Longitudinal	Observational	*Salmonella* spp.	Fecal, individual	Phenotypic, genotypic
Lynch et al., 2018(27)	Ireland	Piglet	Longitudinal	Observational	*Salmonella* spp.	Fecal, individual	Phenotypic
Græsbøll et al., 2017(28)	Denmark	Nursery (4 to 7 weeks)	Randomized trial	Experimental	*E. coli*	Fecal, individual	Phenotypic
Dohmen et al., 2017(29)	The Netherlan ds	Birth, 6, 12, 18 months	Longitudinal	Observational	*E. coli*	Fecal, individual	Phenotypic, genotypic
Pruthvishree et al., 2017(30)	India	1 month, ~2-month, 2-3 month	Cross-sectional	Observational	*E. coli*	Fecal, individual	Phenotypic, genotypic
Fernandes et al., 2016 (31)	Portugal	Piglet, weaner, finisher, sows	Longitudinal	Observational	*Salmonella* spp.	Fecal swab, individual	Phenotypic, genotypic
Cameron-Veas et al., 2016 (32)	Spain	Piglets (7-6 days) and finisher	Trial	Experimental	*E. coli*	Fecal swab, individual	Phenotypic
von Salviati et al., 2014 (33)	Germany	Fattening pig	Longitudinal	Observational	*E. coli*	Fecal, individual and pooled	Phenotypic, genotypic
Hansen et al., 2013 (34)	Denmark	Piglet, nursery, weaner, finisher, sows	Longitudinal	Observational	*E. coli*	Fecal, individual	Phenotypic, genotypic
Quintana-Hayashi et al., 2012 (35)	USA	Nursery, finisher, farrowing	Longitudinal	Observational	*Campylobacte r* spp.	Fecal, individual	Phenotypic
Rosengren et al., 2008 (36)	Canada	Nursery, grower-finisher, and sows	Cross-sectional	Observational	*Salmonella* spp.	Fecal,	Phenotypic
Alali et al., 2008 (37)	USA	Farrow-finish	Longitudinal	Observational	*E. coli*	Composite fresh samples, pooled	Phenotypic
Kobashi, et al., 2008(38)	Japan	4-6 weeks	Trial	Experimental	*E. coli*	Fecal, individual	Phenotypic, genotypic
Dewulf et al., 2007 (39)	Belgium	Nursery, grower, finisher	Longitudinal	Observational	*E. coli*	Fecal, individual	Phenotypic

	Scott et al., 2005 (40)	USA	Farrow-finish	Longitudinal	Observational	*E. coli*	Composite fecal samples	Phenotypic
	Butaye et al., 1999 (41)	Belgium	Pig (piglet, grower/finisher, and sows); poultry (9 and 32 days)	Cross-sectional	Observational	*Entero-coccus* spp.	Fecal, individual	Phenotypic, genotypic
	Mathew et al., 1999(42)	USA	Production pigs (7-63 days) and sows	Longitudinal	Observational	*E. coli*	Fecal swab, individual	Phenotypic
	Moro et al., 1998(43)	USA	Grower, finisher, gilt,and sows	Cross-sectional	Observational	*E. coli*	Fecal, individual	Phenotypic
	Mathew et al., 1998(44)	USA	Piglets (7-63 days) and sows	Longitudinal	Observational	*E. coli*	Fecal, individual	Phenotypic
	Langlois et al., 1988(45)	USA	Weaner, grower, finisher, adults (11-24 months) and sows	Longitudinal	Observational	*E. coli*	Rectal swab, individual	Phenotypic
	Sogaard, 1973(46)	Denmark	Piglet, finisher, sows	Cross-sectional	Observational	*E. coli*	Rectal swab, individual	Phenotypic
Poultry (broiler , layer or turkey, *n*=14)	Baron et al., 2018(47)	France	2, 7 and 77 days	Longitudinal	Observational	*E. coli*	Fecal, individual	Phenotypic, genotypic
	Hume andDunskey, 2017(48)	USA	Day 1-6 weeks (weekly samples)	Trial	Experimental	*Entero-coccus* spp.	Fecal, Cecum	Phenotypic
	Trung et al., 2017(49)	Vietnam	20 weeks, > 20 weeks	Cross-sectional	Observational	*E. coli*	Fecal, individual	Phenotypic, genotypic
	Laube et al., 2013(50)	Germany	1-35 days	Longitudinal	Observational	*E. coli*	Cloacal swab, Individual	Phenotypic, genotypic

	Schwaiger et al., 2013(51)	Germany	21 days and 35 days	Cross-sectional	Observational	*E. coli*	Fecal, floor	Phenotypic, genotypic
	Ozaki et al., 2011(52)	Japan	2 days-50 days	Longitudinal	Observational	*E. coli*	Fresh Dropping	Phenotypic
	da Costa et al., 2009(53)	Portugal	2-33 days	Trial	Experimental	*E. coli and Enterococcus* spp.	Cloacal swab, individual	Phenotypic
	Garcia-Migura et al., 2007(54)	UK	4 days-35 days	Longitudinal	Observational	*Enterococcus* spp.	Fecal, pooled	Phenotypic, genotypic
	Santos et al., 2007(55)	USA	3 weeks and 9 weeks	Cross-sectional	Observational	*Salmonella* spp.	Fecal and litter sample	Phenotypic
	Li et al., 2007(56)	USA	18-72 weeks	Cross-sectional	Observational	*Salmonella* spp.	Fecal, pooled	Phenotypic
	Welton et al., 1998(57)	USA	24 days-130 days	Longitudinal	Observational	*Enterococci* spp.	Cloacal, individual	Phenotypic
	Dubel et al., 1982(58)	USA	1-28 weeks	Cross-sectional	Observational	*E. coli*	Cloacal, individual	Phenotypic
	Nakamura et al., 1982(59)	Japan	5-360 days	Longitudinal	Observational	*E. coli*	Cloacal swab, individual	Phenotypic
	Hinton et al., 1982(60)	UK	1-100 days	Trial	Experimental	*E. coli*	Cloacal swabs, individual	Phenotypic
Dog (*n*=2)	Bang et al., 2017(61)	South Korea	3-6 weeks, 9-28 weeks, 2-6 years and ≥9 years	Cross-sectional	Observational	*Enterococcus* spp.	Fecal, individual	Phenotypic
	Siugzdaite et al., 2017(62)	Lithuania	≤1 year, 1-5 years, 6-10 years, ≥10 years	Cross-sectional	Observational	*Staphylococci* spp.	Fecal, individual	Phenotypic, genotypic

Supplementary Table 4. Quality assessment of the individual studies included in the review (*n*=62)

Species (no. of studies)	Author name (last), year	Country	1. Were the study objectives clearly stated?	2. Were the sampling methods clearly described?	3. Was the sample size calculated?	4. If the study was observational, were inclusion/exclusion criteria specified for subject selection?	5. If the study was experimental, were the groups (treatment and controls) specified?	5.1 Were sampling units randomly assigned to the treatment groups?	6. Were all procedures used in the study specified?	7. Is any bias present in the data collection?	8. Were potential biases and or confounders identified and adjusted or explained (outcome and analysis sections)?
Cattle (*n*=22)	Mir et al., 2018(1)	USA	Yes	Yes	No	Yes	N/A	N/A	Yes	No	Yes
	Pereira et al., 2018(2)	USA	Yes	Yes	No	N/A	Yes	Yes	Yes	No	Yes
	Maynou et al., 2017(3)	Spain	Yes	Yes	No	N/A	Yes	Unclear	Yes	No	Yes
	Adler et al., 2017(4)	Israel	Yes	Yes	No	No	N/A	N/A	Yes	No	Yes
	Ohta et al., 2017(5)	USA	Yes	Yes	No	N/A	Yes	Yes	Yes	No	Yes
	Hutchinson et al., 2017(6)	USA	Yes	Yes	No	Yes	N/A	N/A	Yes	No	Yes
	Catry et al., 2016(7)	Belgium	Yes	Yes	No	Yes	N/A	N/A	Yes	No	Partial
	Pereira et al., 2015(8)	USA	Yes	Yes	Yes	Yes	N/A	N/A	Yes	No	Yes
	Brunton et al., 2014(9)	UK	Yes	Yes	Yes	N/A	Yes	Unclear	Yes	No	Yes
	Mazurek et al., 2013(10)*	Poland	Yes	Yes	No	Partial	N/A	N/A	Yes	Unclear	Partial
	Watson et al., 2012(11)	UK	Yes	Yes	No	Yes	N/A	N/A	Yes	No	Yes
	Alexander et al., 2011(12)	Canada	Yes	Yes	No	N/A	Yes	Unclear	Yes	No	Partial

Species (no. of studies)	Author name (last), year	Country	1. Were the study objectives clearly stated?	2. Were the sampling methods clearly described?	3. Was the sample size calculated?	4. If the study was observational, were inclusion/exclusion criteria specified for subject selection?	5. If the study was experimental, were the groups (treatment and controls) specified?	5.1 Were sampling units randomly assigned to the treatment groups?	6. Were all procedures used in the study specified?	7. Is any bias present in the data collection?	8. Were potential biases and or confounders identified and adjusted or explained (outcome and analysis sections)?
	Sharma et al., 2008(13)	Canada	Yes	Yes	No	N/A	Yes	Yes	Yes	No	Partial
	Berge et al., 2006(14)	USA	Yes	Yes	No	N/A	Yes	Unclear	Yes	No	Yes
	Liebana et al., 2006(15)	UK	Yes	Yes	Yes	Partial	N/A	N/A	Yes	No	Partial
	Hoyle et al., 2006(16)	UK	Yes	Yes	No	Partial	N/A	N/A	Yes	No	Partial
	Donaldson et al., 2006(17)	USA	Yes	Yes	No	Yes	N/A	N/A	Yes	No	Yes
	Berge et al., 2005(18)	USA	Yes	Yes	No	Yes	N/A	N/A	Yes	No	Yes
	Hoyle et al., 2005(19)	UK	Yes	Yes	No	Partial	N/A	N/A	Yes	No	Yes
	Hoyle et al., 2004(20)	UK	Yes	Yes	No	Partial	N/A	N/A	Yes	No	Yes
	Hoyle et al., 2004(21)	UK	Yes	Yes	No	Partial	N/A	N/A	Yes	No	Yes
	Khachatryan et al., 2004(22)	USA	Yes	Yes	No	Partial	N/A	N/A	Yes	No	Partial
Pig (*n*=24)	Ciesinsk et al., 2018(23)	Germany	Yes	Yes	No	N/A	Yes	Yes	Yes	No	Yes
	Randall et al., 2018(24)	UK	Yes	Yes	No	Partial	N/A	N/A	Yes	No	Yes
	Mollenkopf et al., 2018(25)	USA	Yes	Yes	No	No	N/A	N/A	Yes	Unclear	Not Reported
	Cameron-Veas et al., 2018(26)	Spain	Yes	Yes	No	Yes	N/A	N/A	Yes	No	Yes
	Lynch et al., 2018(27)	Ireland	Yes	Yes	No	No	N/A	N/A	Yes	No	Yes

Species (no. of studies)	Author name (last), year	Country	1. Were the study objectives clearly stated?	2. Were the sampling methods clearly described?	3. Was the sample size calculated?	4. If the study was observational, were inclusion/exclusion criteria specified for subject selection?	5. If the study was experimental, were the groups (treatment and controls) specified?	5.1 Were sampling units randomly assigned to the treatment groups?	6. Were all procedures used in the study specified?	7. Is any bias present in the data collection?	8. Were potential biases and or confounders identified and adjusted or explained (outcome and analysis sections)?
	Græsbøll et al., 2017(28)	Denmark	Yes	Yes	No	N/A	Yes	Yes	Yes	No	Yes
	Dohmen et al., 2017(29)	The Netherlands	Yes	Yes	No	Yes	N/A	N/A	Yes	No	Yes
	Pruthvishree et al., 2017(30)	India	Yes	Yes	Yes	No	N/A	N/A	Yes	No	Yes
	Fernandes et al., 2016(31)	Portugal	Yes	Yes	No	Partial	N/A	N/A	Yes	No	Partial
	Cameron-Veas et al., 2016(32)	Spain	Yes	Yes	No	N/A	Yes	Yes	Yes	No	Yes
	von Salviati et al., 2014(33)	Germany	Yes	Unclear	No	Partial	N/A	N/A	Yes	Unclear	Partial
	Hansen et al., 2013(34)	Denmark	Yes	Yes	No	No	N/A	N/A	Yes	No	Yes
	Quintana-Hayashi et al., 2012(35)	USA	Yes	Yes	Yes	Yes	N/A	N/A	Yes	No	Yes
	Rosengren et al., 2008(36)	Canada	Yes	Yes	No	Partial	N/A	N/A	Yes	No	Yes
	Alali et al., 2008(37)	USA	Yes	Yes	No	Yes	N/A	N/A	Yes	No	Yes
	Kobashi, et al., 2008(38)	Japan	Yes	Yes	No	N/A	Yes	Unclear	Yes	No	Yes

Species (no. of studies)	Author name (last), year	Country	1. Were the study objectives clearly stated?	2. Were the sampling methods clearly described?	3. Was the sample size calculated?	4. If the study was observational, were inclusion/exclusion criteria specified for subject selection?	5. If the study was experimental, were the groups (treatment and controls) specified?	5.1 Were sampling units randomly assigned to the treatment groups?	6. Were all procedures used in the study specified?	7. Is any bias present in the data collection?	8. Were potential biases and or confounders identified and adjusted or explained (outcome and analysis sections)?
	Dewulf et al., 2007(39)	Belgium	Yes	Yes	Yes	Yes	N/A	N/A	Yes	No	Yes
	Scott et al., 2005(40)	USA	Yes	Yes	No	Yes	N/A	N/A	Yes	No	Yes
	Butaye et al., 1999(41)‡	Belgium	Yes	Yes	No	Partial	N/A	N/A	Yes	Unclear	Partial
	Mathew et al., 1999 (42)	USA	Yes	Yes	No	Partial	N/A	N/A	Yes	Unclear	Partial
	Moro et al., 1998(43)	USA	Yes	Yes	No	Partial	N/A	N/A	Yes	No	Not Reported
	Mathew et al., 1998(44)	USA	Yes	Yes	No	Partial	N/A	N/A	Yes	Unclear	Yes
	Langlois et al., 1988(45)	USA	Yes	Yes	No	Yes	N/A	N/A	Yes	No	Partial
	Sogaard, 1973(46)	Denmark	Unclear	Unclear	No	No	N/A	N/A	Unclear	Unclear	Not Reported
Poultry (broiler, layer or turkey, *n*=14)	Baron et al., 2018(47)	France	Yes	Yes	No	No	N/A	N/A	Yes	Unclear	Not Reported
	Hume andDunskey, 2017(48)	USA	Yes	Yes	No	N/A	Yes	Unclear	Yes	No	Partial
	Trung et al., 2017(49)	Vietnam	Yes	Yes	No	Yes	N/A	N/A	Yes	No	Yes

Species (no. of studies)	Author name (last), year	Country	1. Were the study objectives clearly stated?	2. Were the sampling methods clearly described?	3. Was the sample size calculated?	4. If the study was observational, were inclusion/exclusion criteria specified for subject selection?	5. If the study was experimental, were the groups (treatment and controls) specified?	5.1 Were sampling units randomly assigned to the treatment groups?	6. Were all procedures used in the study specified?	7. Is any bias present in the data collection?	8. Were potential biases and or confounders identified and adjusted or explained (outcome and analysis sections)?
	Laube et al., 2013 (50) broiler	Germany	Yes	Yes	No	Partial	N/A	N/A	Yes	No	Partial
	Schwaiger et al., 2013(51)	Germany	Yes	Yes	No	Partial	N/A	N/A	Yes	No	Partial
	Ozaki et al., 2011(52) broiler	Japan	Yes	Yes	No	No	N/A	N/A	Yes	No	Yes
	da Costa et al., 2009(53) broiler	Portugal	Yes	Yes	No	N/A	Yes	Unclear	Yes	No	Yes
	Garcia-Migura et al., 2007(54) broiler	UK	Yes	Yes	No	No	N/A	N/A	Yes	No	Not Reported
	Santos et al., 2007(55) turkey	USA	Yes	Yes	No	No	N/A	N/A	Yes	Unclear	Partial
	Li et al., 2007(56) layer	USA	Yes	Yes	No	Partial	N/A	N/A	Yes	No	Partial
	Welton et al., 1998(57) turkey	USA	Yes	Yes	No	Partial	N/A	N/A	Yes	No	Partial
	Dubel et al., 1982(58) turkey	USA	Yes	Yes	No	No	N/A	N/A	Yes	Unclear	Not Reported
	Nakamura et al., 1982(59) broiler and layer	Japan	Unclear	Unclear	No	No	N/A	N/A	Yes	Unclear	Not Reported

Species (no. of studies)	Author name (last), year	Country	1. Were the study objectives clearly stated?	2. Were the sampling methods clearly described?	3. Was the sample size calculated?	4. If the study was observational, were inclusion/exclusion criteria specified for subject selection?	5. If the study was experimental, were the groups (treatment and controls) specified?	5.1 Were sampling units randomly assigned to the treatment groups?	6. Were all procedures used in the study specified?	7. Is any bias present in the data collection?	8. Were potential biases and or confounders identified and adjusted or explained (outcome and analysis sections)?
	Hinton et al., 1982(60) poultry	UK	Yes	Yes	No	N/A	Unclear	Unclear	Yes	No	Not Reported
Dog (*n*=2)	Bang et al., 2017(61)	Korea	Yes	Yes	No	No	N/A	N/A	Yes	No	Yes
	Siugzdaite et al., 2017(62)	Lithuania	Yes	Yes	No	No	N/A	N/A	Yes	Uncl ear	Yes

Yes- quality criteria met; No- quality criteria not met; Partial- not entirely mentioned; Unclear- insufficient information to evaluate quality criteria; N/A- not applicable, *n*-number of studies

Supplementary Figure 1. The average percentages of antimicrobial resistance genes of individual classes in the resistome in fecal samples of production pigs by age (data from *n*=5 studies; the average in each study is plotted).

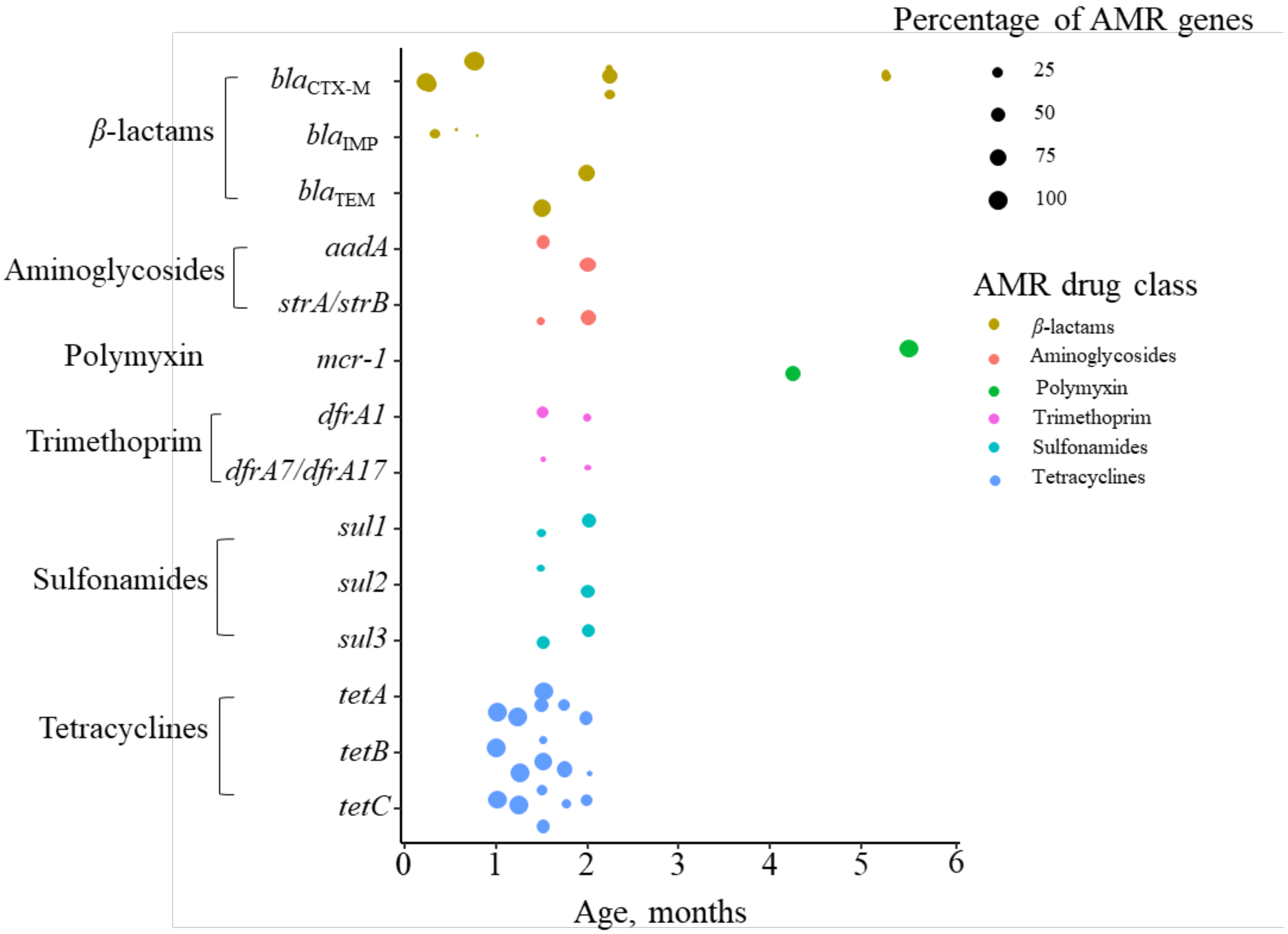

References

1. Mir RA, Weppelmann TA, Teng L, Kirpich A, Elzo MA, Driver JD, et al. Colonization dynamics of cefotaxime resistant bacteria in beef cattle raised without cephalosporin antibiotics. Front Microbiol (2018) 9:500.

2. Pereira RVV, Carroll LM, Lima S, Foditsch C, Siler JD, Bicalho RC, et al. Impacts of feeding preweaned calves milk containing drug residues on the functional profile of the fecal microbiota. Sci Rep (2018) 8:554.

3. Maynou G, Bach A, Terre M. Feeding of waste milk to Holstein calves affects antimicrobial resistance of *Escherichia coli* and *Pasteurella multocida* isolated from fecal and nasal swabs. J Dairy Sci (2017) 100:2682-94.

4. Adler A, Sturlesi N, Fallach N, Zilberman-Barzilai D, Hussein O, Blum SE, et al. Gentamicin- and ciprofloxacin-resistant *Enterobacteriaceae* in cattle farms in Israel: risk factors for carriage and the effect of microbiological methodology on the measured prevalence. Microb Drug Resist (2017) 23:660-5.

5. Ohta N, Norman KN, Norby B, Lawhon SD, Vinasco J, den Bakker H, et al. Population dynamics of enteric *Salmonella* in response to antimicrobial use in beef feedlot cattle. Sci Rep (2017) 7:14310.

6. Hutchinson H, Finney S, Munoz-Vargas L, Feicht S, Masterson M, Habing G. Prevalence and transmission of antimicrobial resistance in a vertically integrated veal calf production system. Foodborne Pathog Dis (2017) 14:711-8.

7. Catry B, Dewulf J, Maes D, Pardon B, Callens B, Vanrobaeys M, et al. Effect of antimicrobial consumption and production type on antibacterial resistance in the bovine respiratory and digestive tract. PLoS One (2016) 11:e0146488.

8. Pereira RV, Siler JD, Cummings KJ, Davis MA, Warnick LD. Effect of heifer-raising practices on *E. coli* antimicrobial resistance and *Salmonella* prevalence in heifer raisers. Epidemiol Infect (2015) 143:3256-65.

9. Brunton LA, Reeves HE, Snow LC, Jones JR. A longitudinal field trial assesing the impact of feeding waste milk containing antibiotic residues on the prevalence of ESBL-producing *Escherichia coli* in calves. Prev Vet Med (2014) 117:403-12.

10. Mazurek J, Pusz P, Bok E, Stosik M, Baldy-Chudzik K. The phenotypic and genotypic characteristics of antibiotic resistance in *Escherichia coli* populations isolated from farm animals with different exposure to antimicrobial agents. Pol J Microbiol (2013) 62:173-9.

11. Watson E, Jeckel S, Snow L, Stubbs R, Teale C, Wearing H, et al. Epidemiology of extended spectrum beta-lactamase *E. coli* (CTX-M-15) on a commercial dairy farm. Vet Microbiol (2012) 154:339-46.

12. Alexander TW, Yanke JL, Reuter T, Topp E, Read RR, Selinger BL, et al. Longitudinal characterization of antimicrobial resistance genes in feces shed from cattle fed different subtherapeutic antibiotics. BMC Microbiol (2011) 11:19.

13. Sharma R, Munns K, Alexander T, Entz T, Mirzaagha P, Yanke LJ, et al. Diversity and distribution of commensal fecal *Escherichia coli* bacteria in beef cattle administered selected subtherapeutic antimicrobials in a feedlot setting. Appl Environ Microbiol (2008) 74:6178-86.

14. Berge AC, Moore DA, Sischo WM. Field trial evaluating the influence of prophylactic and therapeutic antimicrobial administration on antimicrobial resistance of fecal *Escherichia coli* in dairy calves. Appl Environ Microbiol (2006) 72:3872-8.

15. Liebana E, Batchelor M, Hopkins KL, Clifton-Hadley FA, Teale CJ, Foster A, et al. Longitudinal farm study of extended-spectrum beta-lactamase-mediated resistance. J Clin Microbiol (2006) 44:1630-4.

16. Hoyle DV, Davison HC, Knight HI, Yates CM, Dobay O, Gunn GJ, et al. Molecular characterisation of bovine faecal *Escherichia coli* shows persistence of defined ampicillin resistant strains and the presence of class 1 integrons on an organic beef farm. Vet Microbiol (2006) 115:250-7.

17. Donaldson SC, Straley BA, Hegde NV, Sawant AA, DebRoy C, Jayarao BM. Molecular epidemiology of ceftiofur-resistant *Escherichia coli* isolates from dairy calves. Appl Environ Microbiol (2006) 72:3940-8.

18. Berge AC, Atwill ER, Sischo WM. Animal and farm influences on the dynamics of antibiotic resistance in faecal *Escherichia coli* in young dairy calves. Prev Vet Med (2005) 69:25-38.

19. Hoyle DV, Yates CM, Chase-Topping ME, Turner EJ, Davies SE, Low JC, et al. Molecular epidemiology of antimicrobial-resistant commensal *Escherichia coli* strains in a cohort of newborn calves. Appl Environ Microbiol (2005) 71:6680-8.

20. Hoyle DV, Knight HI, Shaw DJ, Hillman K, Pearce MC, Low JC, et al. Acquisition and epidemiology of antibiotic-resistant *Escherichia coli* in a cohort of newborn calves. J Antimicrob Chemother (2004) 53:867-71.

21. Hoyle DV, Shaw DJ, Knight HI, Davison HC, Pearce MC, Low JC, et al. Age-related decline in carriage of ampicillin-resistant *Escherichia coli* in young calves. Appl Environ Microbiol (2004) 70:6927-30.

22. Khachatryan AR, Hancock DD, Besser TE, Call DR. Role of calf-adapted *Escherichia coli* in maintenance of antimicrobial drug resistance in dairy calves. Appl Environ Microbiol (2004) 70:752-7.

23. Ciesinski L, Guenther S, Pieper R, Kalisch M, Bednorz C, Wieler LH. High dietary zinc feeding promotes persistence of multi-resistant *E. coli* in the swine gut. PLoS One (2018) 13:e0191660.

24. Randall LP, Horton RA, Lemma F, Martelli F, Duggett NAD, Smith RP, et al. Longitudinal study on the occurrence in pigs of colistin-resistant *Escherichia coli* carrying mcr-1 following the cessation of use of colistin. J Appl Microbiol (2018) 125:596-608.

25. Mollenkopf DF, Mathys DA, Feicht SM, Stull JW, Bowman AS, Daniels JB, et al. Maintenance of carbapenemase-producing enterobacteriaceae in a farrow-to-finish swine production system. Foodborne Pathog Dis (2018) 15:372-6.

26. Cameron-Veas K, Fraile L, Napp S, Garrido V, Grillo MJ, Migura-Garcia L. Multidrug resistant *Salmonella enterica* isolated from conventional pig farms using antimicrobial agents in preventative medicine programmes. Vet J (2018) 234:36-42.

27. Lynch H, Walia K, Leonard FC, Lawlor PG, Manzanilla EG, Grant J, et al. *Salmonella* in breeding pigs: shedding pattern, transmission of infection and the role of environmental contamination in Irish commercial farrow-to-finish herds. Zoonoses Public Health (2018) 65:e196-206.

28. Graesboll K, Damborg P, Mellerup A, Herrero-Fresno A, Larsen I, Holm A, et al. Effect of tetracycline dose and treatment mode on selection of resistant coliform bacteria in nursery pigs. Appl Environ Microbiol (2017) 83:e00538-17.

29. Dohmen W, Dorado-Garcia A, Bonten MJ, Wagenaar JA, Mevius D, Heederik DJ. Risk factors for ESBL-producing *Escherichia coli* on pig farms: a longitudinal study in the context of reduced use of antimicrobials. PLoS One (2017) 12:e0174094.

30. Pruthvishree BS, Vinodh Kumar OR, Sinha DK, Malik YPS, Dubal ZB, Desingu PA, et al. Spatial molecular epidemiology of carbapenem-resistant and New Delhi metallo beta-lactamase (blaNDM)-producing *Escherichia coli* in the piglets of organized farms in India. J Appl Microbiol (2017) 122:1537-46.

31. Fernandes L, Centeno MM, Couto N, Nunes T, Almeida V, Alban L, et al. Longitudinal characterization of monophasic *Salmonella* typhimurium throughout the pig's life cycle. Vet Microbiol (2016) 192:231-7.

32. Cameron-Veas K, Moreno MA, Fraile L, Migura-Garcia L. Shedding of cephalosporin resistant *Escherichia coli* in pigs from conventional farms after early treatment with antimicrobials. Vet J (2016) 211:21-5.

33. von Salviati C, Friese A, Roschanski N, Laube H, Guerra B, Kaesbohrer A, et al. Extended-spectrum beta-lactamases (ESBL)/AmpC beta-lactamases-producing *Escherichia coli* in German fattening pig farms: a longitudinal study. Berl Munch Tierarztl Wochenschr (2014) 127:412-9.

34. Hansen KH, Damborg P, Andreasen M, Nielsen SS, Guardabassi L. Carriage and fecal counts of cefotaxime M-producing *Escherichia coli* in pigs: a longitudinal study. Appl Environ Microbiol (2013) 79:794-8.

35. Quintana-Hayashi MP, Thakur S. Longitudinal study of the persistence of antimicrobial-resistant *Campylobacter* strains in distinct swine production systems on farms, at slaughter, and in the environment. Appl Environ Microbiol (2012) 78:2698-705.

36. Rosengren LB, Waldner CL, Reid-Smith RJ, Checkley SL, McFall ME, Rajic A. Antimicrobial resistance of fecal *Salmonella* spp. isolated from all phases of pig production in 20 herds in Alberta and Saskatchewan. Can J Vet Res (2008) 72:151-9.

37. Alali WQ, Scott HM, Harvey RB, Norby B, Lawhorn DB, Pillai SD. Longitudinal study of antimicrobial resistance among *Escherichia coli* isolates from integrated multisite cohorts of humans and swine. Appl Environ Microbiol (2008) 74:3672-81.

38. Kobashi Y, Ohmori H, Tajima K, Kawashima T, Uchiyama H. Reduction of chlortetracycline-resistant *Escherichia coli* in weaned piglets fed fermented liquid feed. Anaerobe (2008) 14:201-4.

39. Dewulf J, Catry B, Timmerman T, Opsomer G, de Kruif A, Maes D. Tetracycline-resistance in lactose-positive enteric coliforms originating from Belgian fattening pigs: degree of resistance, multiple resistance and risk factors. Prev Vet Med (2007) 78:339-51.

40. Scott HM, Campbell LD, Harvey RB, Bischoff KM, Alali WQ, Barling KS, et al. Patterns of antimicrobial resistance among commensal *Escherichia coli* isolated from integrated multi-site housing and worker cohorts of humans and swine. Foodborne Pathog Dis (2005) 2:24-37.

41. Butaye P, Devriese LA, Goossens H, Ieven M, Haesebrouck F. Enterococci with acquired vancomycin resistance in pigs and chickens of different age groups. Antimicrob Agents Chemother (1999) 43:365-6.

42. Mathew AG, Saxton AM, Upchurch WG, Chattin SE. Multiple antibiotic resistance patterns of *Escherichia coli* isolates from swine farms. Appl Environ Microbiol (1999) 65:2770-2.

43. Moro MH, Beran GW, Hoffman LJ, Griffith RW. Effects of cold stress on the antimicrobial drug resistance of *Escherichia coli* of the intestinal flora of swine. Lett Appl Microbiol (1998) 27:251-4.

44. Mathew AG, Upchurch WG, Chattin SE. Incidence of antibiotic resistance in fecal *Escherichia coli* isolated from commercial swine farms. J Anim Sci (1998) 76:429-34.

45. Langlois BE, Dawson KA, Leak I, Aaron DK. Effect of age and housing location on antibiotic resistance of fecal coliforms from pigs in a non-antibiotic-exposed herd. Appl Environ Microbiol (1988) 54:1341-4.

46. Sogaard H. Incidence of drug resistance and transmissible R factors in strains of *E. coli* isolated from faeces of healthy pigs. Acta Vet Scand (1973) 14:381-91.

47. Baron S, Le Devendec L, Touzain F, Jouy E, Lucas P, de Boisseson C, et al. Longitudinal study of *Escherichia coli* plasmid resistance to extended-spectrum cephalosporins in free-range broilers. Vet Microbiol (2018) 216:20-4.

48. Hume ME, Donskey CJ. Effect of vancomycin, tylosin, and chlortetracycline on vancomycin-resistant enterococcus faecium colonization of broiler chickens during grow-out. Foodborne Pathog Dis (2017) 14:231-7.

49. Trung NV, Matamoros S, Carrique-Mas JJ, Nghia NH, Nhung NT, Chieu TT, et al. Zoonotic transmission of mcr-1 colistin resistance gene from small-scale poultry farms, Vietnam. Emerg Infect Dis (2017) 23:529-32.

50. Laube H, Friese A, von Salviati C, Guerra B, Kasbohrer A, Kreienbrock L, et al. Longitudinal monitoring of extended-spectrum-beta-lactamase/AmpC-producing *Escherichia coli* at German broiler chicken fattening farms. Appl Environ Microbiol (2013) 79:4815-20.

51. Schwaiger K, Bauer J, Holzel CS. Selection and persistence of antimicrobial-resistant *Escherichia coli* including extended-spectrum beta-lactamase producers in different poultry flocks on one chicken farm. Microb Drug Resist (2013) 19:498-506.

52. Ozaki H, Esaki H, Takemoto K, Ikeda A, Nakatani Y, Someya A, et al. Antimicrobial resistance in fecal *Escherichia coli* isolated from growing chickens on commercial broiler farms. Vet Microbiol (2011) 150:132-9.

53. da Costa PM, Belo A, Goncalves J, Bernardo F. Field trial evaluating changes in prevalence and patterns of antimicrobial resistance among *Escherichia coli* and *Enterococcus* spp. isolated from growing broilers medicated with enrofloxacin, apramycin and amoxicillin. Vet Microbiol (2009) 139:284-92.

54. Garcia-Migura L, Liebana E, Jensen LB, Barnes S, Pleydell E. A longitudinal study to assess the persistence of vancomycin-resistant *Enterococcus faecium* (VREF) on an intensive broiler farm in the United Kingdom. FEMS Microbiol Lett (2007) 275:319-25.

55. Santos FB, Dsouza DH, Jaykus L, Ferket PR, Sheldon BW. Genotypes, serotypes, and antibiotic resistance profiles of *Salmonella* isolated from commercial North Carolina Turkey farms. J Food Prot (2007) 70:1328-33.

56. Li X, Payne JB, Santos FB, Levine JF, Anderson KE, Sheldon BW. *Salmonella* populations and prevalence in layer feces from commercial high-rise houses and characterization of the *Salmonella* isolates by serotyping, antibiotic resistance analysis, and pulsed field gel electrophoresis. Poult Sci (2007) 86:591-7.

57. Welton LA, Thal LA, Perri MB, Donabedian S, McMahon J, Chow JW, et al. Antimicrobial resistance in enterococci isolated from Turkey flocks fed virginiamycin. Antimicrob Agents Chemother (1998) 42:705-8.

58. Dubel JR, Zink DL, Kelley LM, Naqi SA, Renshaw HW. Bacterial antibiotic resistance: frequency of gentamicin-resistant strains of *Escherichia coli* in the fecal microflora of commercial Turkeys. Am J Vet Res (1982) 43:1786-9.

59. Nakamura M, Yoshimura H, Koeda T, Sato S. Fluctuation of drug-resistant *Escherichia coli* strains in adult chickens. Nihon Juigaku Zasshi (1982) 44:397-402.

60. Hinton M, Al-Chalaby ZA, Allen V, Linton AH. The persistence of drug resistant *Escherichia coli* in the intestinal flora of healthy broiler chicks. J Hyg (Lond) (1982) 89:269-78.

61. Bang K, An JU, Kim W, Dong HJ, Kim J, Cho S. Antibiotic resistance patterns and genetic relatedness of *Enterococcus faecalis* and *Enterococcus faecium* isolated from military working dogs in Korea. J Vet Sci (2017) 18:229-36.

62. Siugzdaite J, Gabinaitiene A. Methicillin-resistant coagulase-negative staphylococci in healthy dogs. Vet Med (2017) 62:479-87.
